# Cross-frequency coupling in real and virtual brain networks

**DOI:** 10.3389/fncom.2013.00078

**Published:** 2013-07-03

**Authors:** Viktor Jirsa, Viktor Müller

**Affiliations:** ^1^Institut de Neurosciences des Systèmes, Faculté de Médecine, Aix-Marseille Université, Inserm UMR1106Marseille, France; ^2^Center for Lifespan Psychology, Max Planck Institute for Human DevelopmentBerlin, Germany

**Keywords:** cross-frequency coupling, the virtual brain, bispectrum, bicoherence, simulation, resting state, full brain model, connectome

## Abstract

Information processing in the brain is thought to rely on the convergence and divergence of oscillatory behaviors of widely distributed brain areas. This information flow is captured in its simplest form via the concepts of synchronization and desynchronization and related metrics. More complex forms of information flow are transient synchronizations and multi-frequency behaviors with metrics related to cross-frequency coupling (CFC). It is supposed that CFC plays a crucial role in the organization of large-scale networks and functional integration across large distances. In this study, we describe different CFC measures and test their applicability in simulated and real electroencephalographic (EEG) data obtained during resting state. For these purposes, we derive generic oscillator equations from full brain network models. We systematically model and simulate the various scenarios of CFC under the influence of noise to obtain biologically realistic oscillator dynamics. We find that (i) specific CFC-measures detect correctly in most cases the nature of CFC under noise conditions, (ii) bispectrum (BIS) and bicoherence (BIC) correctly detect the CFCs in simulated data, (iii) empirical resting state EEG show a prominent delta-alpha CFC as identified by specific CFC measures and the more classic BIS and BIC. This coupling was mostly asymmetric (directed) and generally higher in the eyes closed (EC) than in the eyes open (EO) condition. In conjunction, these two sets of measures provide a powerful toolbox to reveal the nature of couplings from experimental data and as such allow inference on the brain state dependent information processing. Methodological advantages of using CFC measures and theoretical significance of delta and alpha interactions during resting and other brain states are discussed.

## Introduction

“Almost all biological systems exhibit significant non-linear behavior” (Sigl and Chamoun, 1994). The non-linear nature is imminent in electrophysiological brain activity as measured by Electroencephalography (EEG) or Magnetoencephalography (MEG; Elbert et al., 1994; Birbaumer et al., 1995; Müller et al., 2003a; Allefeld et al., 2009) and results in characteristics such as multistability, bifurcations, deterministic chaos, and multiscale behaviors. Even at rest (in the absence of an explicit task), the human brain shows temporally coherent activity (Deco et al., 2008, 2009; Ghosh et al., 2008) of a surprising degree of complexity. This so-called “resting state” activity and its underlying coupling dynamics can be captured at different scales (from a single cortical area to multiple cortical areas and whole brain dynamics) and frequencies using both neuroimaging techniques (fMRI and PET) and EEG/MEG recordings (Biswal et al., 1995; Greicius et al., 2003; Müller et al., 2003a,b; Damoiseaux et al., 2006; Venables et al., 2009). Moreover, the EEG (and MEG) is a complex signal containing different frequency components interacting with each other. Classic power spectral analyses based on (fast) Fourier Transform (FFT) or different time-frequency transforms (e.g., wavelet, Hilbert, or Gabor transform) display amplitude modulations within the defined frequencies across time. Corresponding complex transformations of the signal provide information about phase changes but they fail identifying the relationships among different frequencies or frequency components. However, it is the cross-frequency coupling (CFC) between different frequency bands that has been hypothesized to be the carrier mechanism for the interaction of local and global processes and hence being directly related to the integration of distributed information. In the early 1960s, bispectral analysis was first introduced by geophysicists (cf. Sigl and Chamoun, 1994) to study the interfrequency coupling of geophysical signals. These algorithms have then been used also in neurosciences, especially during the last decade in the EEG literature (Sigl and Chamoun, 1994; Witte et al., 2000; Hagihira et al., 2001; Schack et al., 2001a,b, 2002; Miller et al., 2004; Isler et al., 2008).

## Different types of cross-frequency coupling (CFC)

Recently, Jensen and Colgin (2007) described different forms of cross-frequency interactions: (i) power to power, (ii) phase to phase, (iii) phase to frequency, and (iv) phase to power. There is an increasing evidence that the last type of CFC, so-called phase-amplitude modulation, occurs very often and was found both in animals and humans in the entorhinal and prefrontal cortices, in the hippocampus, and distributed cortical areas (Mormann et al., 2005; Cohen, 2008; Osipova et al., 2008; Tort et al., 2008, 2009, 2010; Cohen et al., 2009a,b; Colgin et al., 2009; Axmacher et al., 2010a,b; Voytek et al., 2010). According to this CFC, “gamma oscillations might emerge at a particular phase of the theta cycle and thereby recruit cell assemblies involved in processing at that time” (Jensen and Colgin, 2007). Bruns and Eckhorn (2004) investigated also cross-frequency *amplitude* modulations by means of envelope-to-envelope and envelope-to-signal correlations using subdural electrodes in epileptic patients during a visual delayed-match-to-sample task. They found a pronounced task-related increase of the gamma-delta envelope-to-signal correlation (with a correlational delay of 40 ms) between superior and inferior occipital visual areas possibly reflecting a short-term memory encoding process. In contrast, envelope-to-envelope correlation showed event-, but not task-related changes of intra-areal and no changes of inter-areal coupling (Bruns and Eckhorn, 2004). De Lange et al. (2008) investigated cross-frequency amplitude correlation during motor imagery and found interactions between central and precentral alpha/beta oscillations and occipito-parietal gamma oscillations. In addition to the mentioned above cross-frequency (CF) modulations, Witte et al. (2008) described two more CFC types: envelope to frequency and frequency to frequency. In the data-based EEG burst simulations using coupled Duffing oscillators, the authors (Witte et al., 2008) found strong envelope-envelope and envelope-frequency CFC in the delta (0.5–2.5 Hz) and the alpha (7–11 or 8–12 Hz) bands and quadratic coupling using bicoherence (*BIC*) between delta and alpha bands.

## Theta-gamma oscillatory coupling

Neurophysiological evidence suggests that oscillations in theta and gamma band are simultaneously modulated during perception and memory (Jensen and Colgin, 2007; Colgin et al., 2009; Tort et al., 2009). Recently, more and more evidence suggests that corresponding CFC between these frequency bands plays a crucial role in this and other processes, e.g., neuronal computation, communication, and learning (Schack et al., 2002; Schack and Weiss, 2005; Canolty et al., 2006; Jensen and Colgin, 2007; Cohen, 2008; Tort et al., 2008, 2009; Doesburg et al., 2009; Canolty and Knight, 2010; Kendrick et al., 2011). In the study of Schack et al. (2002), increased power in the theta and the gamma frequency bands was accompanied by strong phase coupling by means of cross- *BIC* between theta frequency at Fz and gamma frequency at F3 and Fp1, respectively, for memorizing number words. The suggestion that this is an amplitude modulation of gamma oscillations by slow frequency oscillations (e.g., theta) was supported by coherence analysis between the envelope of gamma frequencies and the raw EEG. In another study of Schack and Weiss (2005), the CFC between theta and gamma oscillations was investigated using *n*:*m* phase synchronization algorithms based on Gabor expansion function. Besides the higher spectral power, phase locking and 1:1 phase synchronization measured by phase locking index (PLI) and phase coherence (PC) in both the theta and the gamma frequency bands, successful encoding of nouns was also accompanied by increased CFC or 1:6 phase synchronization at selected electrodes (within the time interval of 200–250 ms) and between them (within the time intervals of 250–350 and 400–500 ms). A phase to power CFC between theta and gamma oscillations was also reported in epilepsy patients during a continuous word recognition paradigm in the rhinal cortex and hippocampus. Interestingly, the theta-gamma CFC in the rhinal cortex was more pronounced for correct rejections than for hits, while this CFC pattern in the hippocampus was inversely more pronounced for hits than for correct rejections (Mormann et al., 2005). Using intracranial recordings in human epilepsy patients, Axmacher et al. (2010a) showed (i) that simultaneous maintenance of multiple items in working memory is accompanied by theta-gamma phase-amplitude CFC in the hippocampus, and (ii) that maintenance of an increasing number of items is associated with modulation of beta/gamma power by lowering theta frequency phase. In other words, modulating influence of the lower theta phase on the beta/gamma activity provides for higher working memory load. Recently, Belluscio et al. (2012) found that theta-gamma phase-amplitude modulation in the CA1 region of rat hippocampus was accompanied by theta-gamma phase-phase modulations, at least for slow (30–50 Hz) and midfrequency (50–90 Hz) gamma oscillators.

## Delta-theta/delta-alpha oscillatory coupling and other CFCs

Besides the cross low-frequency/high-frequency coupling (e.g., theta-gamma), there is evidence (Lakatos et al., 2005; Schack et al., 2005; Cohen, 2008; Isler et al., 2008) that CFC exists also between the low-frequency bands (e.g., delta-theta, delta-alpha, and theta-alpha). Isler et al. (2008) reported increase in power and coherence in the delta band elicited by novel sounds in an auditory novelty oddball task accompanied by CFC measured by *BIC* for delta-theta (1:3) and delta-alpha (1:4) relationships in widespread fronto-central, right parietal, temporal, and occipital regions. At the same time, globally synchronized delta oscillations were phase coupled in terms of cross-bicoherence (*cBIC*) to theta oscillations in central regions and to alpha oscillations in right parietal and posterior regions. Using CF m:n phase synchronization index (PSI), Schack et al. (2005) found an increase in upper alpha-theta phase synchronization between right posterior and left anterior sites in a memory scanning task. The authors suggested that this CFC reflects the interplay between the central executive functions (theta) and the reactivation of long-term memory codes in short-term memory (upper alpha). In a competitive decision-making task Cohen et al. (2009b) found that alpha and beta amplitude in human medial frontal cortex was modulated by delta and theta phase; the strength of this modulation differed also between losses and wins, suggesting that this CF phase-amplitude coupling might reflect a coding mechanism of feedback valence information.

Recently, Lakatos et al. (2005) introduced a hypothesis about the “hierarchical” organization of EEG oscillations suggesting that the amplitude of the oscillations at characteristic frequency is modulated by the oscillatory phase at lower frequency. In particular, they found that delta (1–4 Hz) phase modulates theta (4–10 Hz) amplitude, and theta modulates gamma (30–50 Hz) amplitude in primary auditory cortex of awake macaque monkeys (Lakatos et al., 2005). Interestingly, in full-term newborns, *n:m* phase synchronization between two delta rhythms (1–1.5 and 3.5–4.5 Hz) was reported (Wacker et al., 2010).

Osipova et al. (2008) reported also about phase to power CFC between alpha and gamma MEG oscillations during rest with eyes closed (EC). Interestingly, there was no peak in the gamma frequency band and gamma activity was only evident when studied in relation to the alpha phase. In another MEG study (Palva et al., 2005), marked cross-frequency *n*:*m* phase synchrony was found among oscillations with frequencies from 3 to 80 Hz. In particular, enhanced CF phase synchrony among alpha, beta, and gamma frequency oscillations was present during continuous mental arithmetic tasks demanding the retention and summation of items in the working memory. This enhancement of CF phase synchrony is considered as a candidate mechanism for the integration of spectrally distributed processing (Palva et al., 2005). Gamma amplitude modulation (40–80 Hz) by the phase of the alpha band oscillations (8–12 Hz) was found in the nucleus accumbens of human patients undergoing deep brain stimulation surgery during a simple reward task (Cohen et al., 2009a). Recently, it was provided evidence that posterior alpha oscillations (8–13 Hz) constitute a mechanism for prioritizing and ordering unattended visual input. This mechanism is suggested to be based on alpha-gamma phase-amplitude CFC, whereby gamma amplitude-modulated activity that is phase locked to the alpha-phase keeps competing unattended representations apart in time (Jensen et al., 2012). In a study with implanted subdural electrocorticographic grids in two patients with intractable epilepsy performing different visual and non-visual tasks (Voytek et al., 2010), it was found that high gamma amplitude (80–150 Hz) is modulated in a non-visual task by anterior frontal theta phase and in a visual task by the occipital alpha phase. Thus, the modulation of high gamma activity through theta and alpha phase varied in these patients as a function of brain area and task modality. The fact that high-frequency power can be modulated by the phase of multiple brain rhythms simultaneously provide evidence that CFC may constitute a mechanism for selection between communicating cell assemblies (Canolty and Knight, 2010; Voytek et al., 2010).

## Information flow within and between cell assemblies

Beginning 1920s, Karl Lashley started with his historical works about memory traces (engrams) in cerebral cortex and showed that distribution of active and inactive synapses can be an evidence for learning processes (Lashley, 1924, 1931). Lashley's student, D. O. Hebb, developed his so-called Theory of Cell Assemblies (Hebb, 1949) on the basis of the Lorente de No's concept of reverberatory circuits. These circuits have been considered as the mechanism of activity maintenance after the stimulus effect was reversed. Besides the properties of fast firing and excitation persistence, cell assemblies can be considered as “closed systems” oscillating synchronously at different frequencies with strong information flow within each cell assembly and much smaller information flow between them. In order to prevent “the transition from an ‘Einfall’ to an ‘Anfall’ (transition from an idea to a seizure) in an excitatory neuronal network or, as Braitenberg poetically states, to ‘discover and isolate ideas …’, reinforce ideas, ‘and keep them separately’ (Braitenberg and Schüz, 1991, p. 205)” (cited by Birbaumer et al., 1995, p. 451), cell assemblies must possess their own automatic threshold control. Separate cell assemblies communicate with each other to integrate single information flows and ideas into a common network or thinking process. In terms of dynamic systems, these metaphoric descriptions can be rephrased as convergence or divergence of flows in state space allowing for a full dynamic description (Jirsa and Kelso, 2005; Perdikis et al., 2011). One of the mechanisms underlying such an integration or communication between different cell assemblies might be the CFC, allowing accurate timing between different oscillatory rhythms, selective and dynamic control of distributed functional cell assemblies (cf. Canolty et al., 2010), and promotion of different dimensions of brain integration (Varela et al., 2001; Buzsáki and Draguhn, 2004; Allen et al., 2011).

## Cross-frequency measures

### Bispectrum and bicoherence

Bispectral analysis is an advanced signal processing technique based on high-order statistics (HOS). This technique, analyzing multiplicative connections between two rhythms, generating a third frequency component, and quantifying quadratic non-linearities and deviation from normality, may be used to investigate non-linearities within the signal [in the case of bispectrum (*BIS*) or bicoherence (*BIC*)] or between the signals [in the case of cross-bispectrum (*cBIS*) or cross-bicoherence (*cBIC*)] arising from inter-frequency coupling within and between the signals, respectively (Sigl and Chamoun, 1994; Schack et al., 2002; Miller et al., 2004; Isler et al., 2008).

*BIS* is a higher-order extension of power spectral estimation. A conventional power spectrum decomposes the power of a time series over frequency. In contrast, the *BIS* decomposes the third moment (skewness) of a time series over frequencies. Specifically, BIS estimates the relationship between oscillatory components of the signal or more precisely between the oscillations at two basic frequencies, *f*_1_ and *f*_2_, and a harmonic component at the frequency *f*_1_ + *f*_2_. The *BIS* incorporates both phase and power information, and can be calculated for each frequency triplet (*f*_1_, *f*_2_, and *f*_1_ + *f*_2_). The estimated *BIS* can be used to detect asymmetric non-linearities in a time series and to detect phase coupling between frequency components. But because the magnitude of the *BIS* is influenced by the amplitude of the signal, it is not a pure measure of the degree of phase coupling. Instead, normalized *BIS* called *BIC* can be used for this purpose. *BIC* is defined as a ratio of the *BIS* to the square root of the real triple product computed from the power spectrum (see Methods); thus, the ratio is independent of signal amplitude and *BIC* is, therefore, considered as a pure measure of the degree of phase coupling (Sigl and Chamoun, 1994; Schack et al., 2002). *BIC* ranges between 0 and 1, with 0 indicating no phase coupling and 1 indicating complete phase coupling between two frequency components. All this is also true for cross-*BIS* (*cBIS*) and cross-*BIC* (*cBIC*) with the difference that the CFC in this case is estimated between two signals.

### Other specific CFC estimates

**Figure 1:**
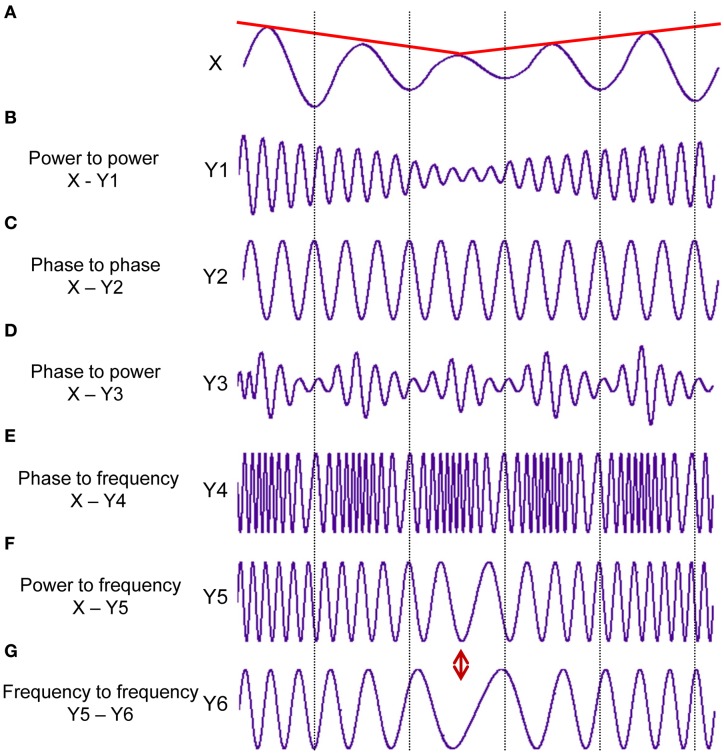
**Different types of the cross-frequency coupling. (A)** Signal *X* at a given constant frequency fluctuating in the amplitude over time (red line). **(B)** Power to power CFC: Signal *Y1* at about 5 times higher frequency than in the signal *X* showing slow amplitude modulations over time like signal *X* (red line). **(C)** Phase to phase CFC: Signal *Y2* showing 3:1 phase to phase coupling with signal *X*. One oscillation period of signal *X* corresponds to three periods of signal *Y2*. **(D)** Phase to power CFC: Signal *Y3* with fast amplitude modulations, which are related or coupled with the phase of the signal *X*. **(E)** Phase to frequency CFC: Signal *Y4* with frequency modulations, which are coupled with phase changes of signal *X*. **(F)** Power to frequency CFC: Signal *Y5* with frequency modulations, which are coupled with the slow amplitude modulations of signal *X* (red line). **(G)** Frequency to frequency CFC: Signal *Y6* with slower frequency modulations than in the signal *Y5*. The different types of CFC are not mutually exclusive (ref. Jensen and Colgin, 2007). It can be seen for instance, that slow amplitude modulations of Signal *X* are coupled not only with the amplitude changes of the signal *Y1* but also with frequency changes of signals *Y5* and *Y6*, which are at the same time coupled in their frequency modulations.

Taking into account the main characteristics of the signal(s), six different CFC measures may be obtained: (i) power to power, (ii) phase to phase, (iii) phase to power, (iv) power to frequency, (v) phase to frequency, and (vi) frequency to frequency (cf. Jensen and Colgin, 2007). These CFC measures reflect different aspects of CFC, which are schematically presented in Figure 1. Together with *BIC* and *BIS*, these CFC measures give a relatively complete picture about cross-frequency interdependencies within and between the signals.

*Power to power CFC* indicates how amplitude modulations in one frequency depend on amplitude modulations in the other frequency (compare envelopes of signals X and Y1 in Figures 1A,B, respectively). This type of CFC was developed to investigate coupling between different high-frequency oscillations (e.g., beta, gamma) showing similar (low-frequency) amplitude modulations (Bekisz and Wrobel, 1999; Bruns et al., 2000; Bruns and Eckhorn, 2004). The advance of this technique consist in detecting not only coupling between different frequencies but also coupling within the same frequency, which could not be found using conventional coherence measures because of large temporal or phase jitter (Bruns et al., 2000).

*Phase to phase CFC* is a measure for *n*:*m* synchronization and shows the degree of the true phase coupling between the frequencies within and between the signals (compare phases of signals X and Y2 in Figures 1A,C, respectively; the phase relation is 1:3 in this case). This type of CFC is a pure phase coupling measure and is amplitude-independent. This so-called *n*:*m* synchrony indicates phase locking on *n* cycles of one oscillation to *m* cycles of another oscillation (Rosenblum et al., 1996; Tass et al., 1998). As mentioned by Rosenblum and colleagues (Rosenblum et al., 1996), “the phenomenon of phase synchronization is a characteristic feature of autonomous continuous-time system.” In this regard, the phase synchronization and especially the cross-frequency phase synchronization is an excellent candidate for neural temporal coding supporting dynamic information flow in the brain.

*Phase to power CFC* reflects amplitude modulations in one frequency (normally in the high frequency, e.g., signal Y3 in Figure 1D) dependent on the phase of the other frequency (low frequency, e.g., signal X in Figure 1A). Besides the previous CFC, the phase to power coupling is the most commonly used or the best-studied type of coupling (e.g., the mentioned above theta-gamma CFC). Like the cross-frequency phase synchronization, also the phase to power CFC is a good candidate for the neural temporal coding, with the difference that (and especially when) specific amplitude modulations (normally high-frequency modulations, e.g., gamma) of one oscillation take place in specific time windows or phase state of another oscillation (normally low-frequency oscillation, e.g., theta). Moreover, in terms of neural coding, it can be considered as an integration mechanism between the two types of coding (i.e., rate and temporal code), whilst amplitude modulations represent the rate coding and phase course reflects the temporal coding. Furthermore, in some cases (e.g., Hebbian learning), when firing rate decreases or will be replaced by temporally more accurate firing, it will be considered as transforming a rate code into a temporal code (Mehta et al., 2002). As recently shown (Jensen et al., 2012), the phase of ongoing alpha oscillation, inhibiting neuronal processing, modulates neuronal excitability in form of gamma activity in the way that neural firing and corresponding gamma amplitude modulations occur during the falling phase of alpha oscillation. So, it is assumed that “alpha activity provides a clocking mechanism that controls neuronal processing reflected by activity in the gamma band” (Jensen et al., 2012, p. 200).

*Power to frequency CFC* indicates changes in the frequency induced by changes in the amplitude of the signal or envelope (compare envelope of signal X and frequency modulations of signal Y4 in Figures 1A,E, respectively). This type and also the next two types of CFC, where frequency is one of the interaction components, have been poorly investigated until recently. We can only refer to the study of Witte et al. (2008), where this type of CFC was addressed. Instantaneous frequency (IF) is defined in this case as the phase changes in time (see Methods for details). As observable in Figure 1, these signal modulations or coupling types cannot be detected by other CFC measures but they seem to play a crucial role in the systems with high dynamic changes, which can only be explained by frequency/phase entrainment (Witte et al., 2008). Furthermore, amplitude-frequency modulations can be attributed to so-called auto resonance, when change in the drive frequency causes a corresponding change in the oscillation amplitude, which leads to entrainment or sustained phase locking of the driving and the oscillator frequency (Witte et al., 2008). It is also well known that the frequency modulation as compared with the amplitude modulation allows a higher dynamic range of the information signal and is less susceptible to interference or disturbances. However, these aspects of the information processing have not been investigated well in neurosciences until now.

*Phase to frequency CFC* indicates changes in the frequency induced by the phase of the signal (compare phase of signal X and frequency modulations of signal Y5 in Figures 1A,F, respectively). This type of CFC can also have a high scientific relevance adding further important information regarding CF interaction as these aspects have not been studied until now.

*Frequency to frequency CFC* reflects changes in the one frequency range induced through changes in the other frequency range (compare frequency modulations of signals Y5 and Y6 in Figures 1F,G, respectively). Also this type of CFC can provide additional information about interacting systems or cell assemblies and extend our understanding of the cross-frequency neural communication.

## Simulation data: reduced oscillators from full realistic brain networks

To test and validate the CFC measures, we phenomenologically derive the mathematical form of generic oscillator equations from a full brain network. The purpose of this derivation is to motivate the influences of certain structural and architectonic elements of full brain networks on generic oscillator equations and then solely discuss the latter in the context of our simulations. Full brain network models comprise neural population models at each network node modeling the activity of a brain region. The nodes are connected via large-scale connectivity matrices, the so-called connectome. Traversing the scale of description from the full brain network to reduced oscillator models will impose constraints on the choice of the parameters. The parameters will in general not be freely adjustable, but will interdepend and lie on so-called manifolds in parameter spaces. The manifolds are hypersurfaces that constrain the possible combinations of parameters. For the purposes of this study, we keep the parameters unconstrained and choose a specific parameter combination capable of generating a dynamic behavior regarding its CFC. A phenomenological modeling of this sort will allow us to introduce noise into the model system and generate more realistic situations, which we can then put to the test using our battery of CFC measures. We lose, however, the possibility to interpret the parameters in the reduced model physiologically. Marder and Goaillard (2006) pointed out that such parameter manifolds may comprise surprisingly large ranges and may be shaped in a complex manner. As a consequence, sometimes the use of average parameters may not only be a bad approximation, but may indeed provide incorrect results, since the average parameter values may actually not be on the constraining manifold and hence display a different dynamic behavior (than those on the manifold). This evidence demonstrates that the fitting of models for specific parameters is only of limited value, rather the determination of parameter ranges and the respective manifolds in the parameter spaces is asked for. In the subsequent discussion, we will demonstrate how the phenomenological oscillator models are motivated from the large-scale brain network. We then freely change the parameters to generate various types of CFC relevant for the discussion in this article.

The network nodes of a full brain network are neural mass models typically derived from neuron interactions using a mean-field approach. Common assumptions in mean-field modeling are that explicit structural features or temporal details of neuronal networks (e.g., spiking dynamics of single neurons) are irrelevant for the analysis of complex mesoscopic dynamics, and the emergent collective behavior is only weakly sensitive to the details of individual neuron behavior (Breakspear and Jirsa, 2007). Basic mean field models capture changes of the mean firing rate (Brunel and Wang, 2003), whereas more sophisticated mean field models account for parameter dispersion in the neurons and the subsequent richer behavioral repertoire of the mean field dynamics (Assisi et al., 2005; Stefanescu and Jirsa, 2008, 2011; Jirsa and Stefanescu, 2011). These approaches demonstrate a relatively new concept from statistical physics that macroscopic physical systems obey laws that are independent of the details of the microscopic constituents they are built of (Haken, 1983). These and related ideas have been exploited in neurosciences (Kelso, 1995; Buzsaki, 2006). Thus, our main interest lies in deriving the mesoscopic laws that drive the observed dynamical processes at the macroscopic scale in a systematic manner.

In the framework of “The Virtual Brain” (TVB, www.thevirtualbrain.org) we develop full brain network models by incorporating biologically realistic large scale coupling of neural populations at salient brain regions that is mediated by long-range neural fiber tracts as identified with diffusion tensor imaging (DTI) based tractography together with mean-field models as local node models. Various mean-field models are available in TVB, reproducing typical features of mesoscopic population dynamics (see Sanz Leon et al., 2013, for details). Each network node is governed by its own intrinsic population dynamics in interaction with the dynamics of all other network nodes. This interaction happens through the connectivity matrix via specific connection weights obtained from DTI and time delays due to signal transmission delays. The general evolution equation (see Jirsa, 2009) captures these architectonic features through a stochastic integral-differential equation of a network of connected neural populations derived from mean field approaches using coupled neurons. Noise plays a crucial role for the brain dynamics, and hence for brain function (McIntosh et al., 2010), and is typically introduced additively where the type of noise and its spatial and temporal correlations can be specified independently. Though the evolution equation in TVB captures all the relevant features of connectivity and neural mass modeling, it is far from obvious how to systematically control the actual nature of the CFCs systematically and independently within the framework of TVB. Different network parameter manipulations will affect various forms of CFC in a non-unique manner. Hence, to allow for a systematic discussion of a generative evolution equation with and without noise, but still sufficiently motivated by the original TVB philosophy, we follow the steps of Jirsa (2009), where the full brain network equation comprised full local connectivity, but only one long-range two-point connection with signal transmission delay. Jirsa (2009) considered the effects of the time delay explicitly, which we will though ignore here (equivalent to the assumption of the time delay being small with regard to the time scale of the oscillator dynamics). Jirsa (2009) reduced this model system to two non-linearly coupled oscillators with state variables, *x*_1_(*t*) and *x*_2_(*t*) and performed a linear stability analysis of their equilibrium state. Here, we are interested in their non-linear oscillatory behavior and hence take a modified approach as follows: we preserve all non-linearities in the original non-linear oscillator equations as described by Jirsa (2009) and formally decompose the state variables *x*_1_(*t*) and *x*_2_(*t*) into their amplitudes, *r*_1_(*t*) and *r*_2_(*t*) and phases, φ_1_(*t*) and φ_2_(*t*). Then we perform a Taylor decomposition in the amplitudes and a Fourier decomposition in the phases to obtain the following set of equations:

$${\dot{r}}_{i}(t)=r_{i}(t)-\sum_{j}a_{ij}r_{j}^{2}(t)r_{i}(t)-\sum_{j,\;n,\;m}b_{ijnm}\sin(n\varphi_{i}(t)-m\varphi_{j}(t))r_{i}(t)$$

(1) $${\dot{\varphi }}_{i}(t)=\omega_{i}(1+\sum_{j}c_{j}r_{j}(t))-\sum_{j,\;n,\;m}d_{ijnm}\sin(n\varphi_{i}(t)-m\varphi_{j}(t))$$

where we kept only the leading orders of terms in phase and amplitude as relevant for our discussion. Here, we used a general formulation of the coupled phase-amplitude equations for an arbitrary number N of oscillators with indices *i*, *j* = 1, …, *N* and where *n*, *m* = 0, …, are the orders of the Fourier expansions of the fully non-linear oscillator equations in (Jirsa, 2009); ω_*i*_ is the oscillation frequency and *a*_*ij*_, *b*_*ijnm*_, *c*_*j*_, and *d*_*ijnm*_ are constant coefficients. All parameter values are defined in the Methods. The latter coefficients can be expressed in principle through the architectural elements including connectivity, sigmoidal response function and local neural node dynamics, but these expressions will be generally complicated and not unique. As we here choose the parameters freely (see Methods below), all terms absent in Equation 1 can be considered to have been set to zero. In the stochastic version, the above equations contain linearly added white Gaussian noise. We used the Euler Maruyama algorithm to solve the equations (Kloeden and Platen, 1992).

## Methods

### Simulation data

To test the validity and performance of the different types of CFC, we applied different CFC measures to simulated data. The parameter choice for each instance of CFC was motivated only by considerations from non-linear dynamics theory (see for instance Strogatz, 1994) with the intent to maximize a desired CFC effect. No consideration was given to potential co-dependencies of coefficients in Equation 1 on the same structural substrate (connectivity, or others). The eigenfrequencies for all simulations were ω_1_ = 32π and ω_2_ = 32π /5. The other parameter choices have been selectively made for the various forms of CFC:

- *Power to power*: amplitude modulations in one frequency depend on amplitude modulations in the other frequency. Parameters: *a*_11_ = *a*_12_ = *a*_22_ = 1, all others are zero.
- *Phase to phase*: phases are directly interdependent. Parameters: *a*_11_ = *a*_22_ = 1, *d*_1215_ = −170, *d*_2151_ = −42, all others are zero.
- *Phase to power*: amplitude modulations in the high frequency depend on the phase of the small frequency. Parameters: *a*_11_ = *a*_22_ = 1, *b*_1201_ = −15, all others are zero.
- *Power to frequency*: changes in the frequency induced by changes in the amplitude of the signal or envelope. Parameters: *a*_11_ = *a*_22_ = 1, *c*_1_ = 0.9, all others are zero.
- *Phase to frequency*: changes in the frequency are induced by the phase of the signal. Parameters: *a*_11_ = *a*_22_ = 1, *d*_1201_ = −22π, all others are zero.

**Figure 2:**
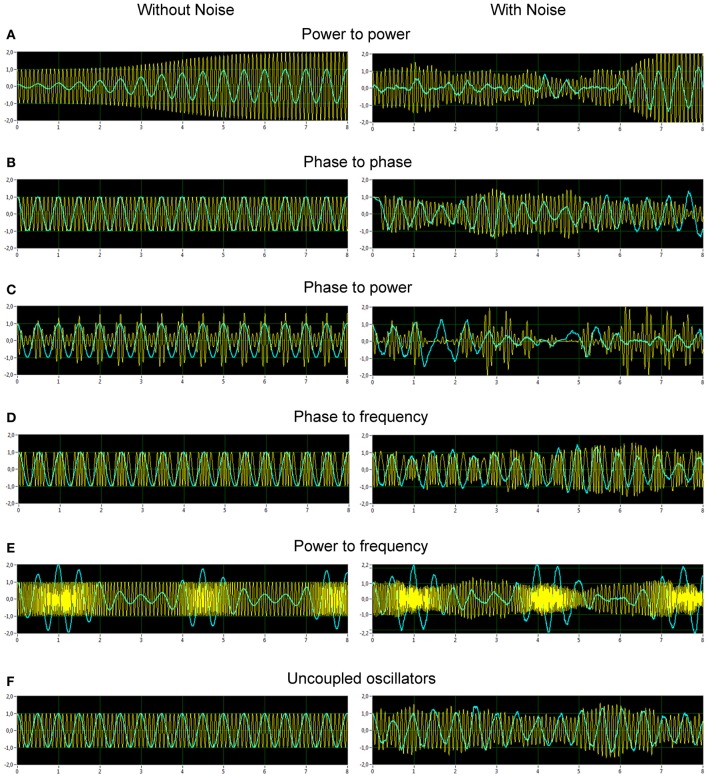
**Simulated data representing different types of CF interactions in absence and in presence of noise. (A)** Power to power modulation. **(B)** Phase to phase modulation. **(C)** Phase to power modulation. **(D)** Phase to frequency modulation. **(E)** Power to frequency modulation. **(F)** Uncoupled oscillators. The left column displays the time series in absence of noise, the right column in presence of noise.

Figure 2 displays the two oscillators of the five CFC types and a case of uncoupled oscillators. The left column illustrates the time series in absence of noise, the right column in presence of noise. Frequency to frequency coupling was omitted in this presentation, since it has a particularity compared to the other forms of coupling, that is the notion of frequency is not in a unique relation with the amplitudes, *r*_1_(*t*) and *r*_2_(*t*), and phases, φ_1_(*t*) and φ_2_(*t*). The frequency can be computed in various ways and will introduce a new state variable, which, by definition, will change the nature of the dynamic system and does not fall into the framework we have developed in Equations 1. For these reasons we choose to omit the discussion of this coupling here.

### EEG recording during resting state

#### Participants

All participants were volunteers and were recruited through announcements on Saarland schools (Gymnasiums) and Saarland University. For participation in the study, all subjects were paid 7.5 Euro per hour. All the subjects were right-handed, had no reported history of head or neurological disorders, and none were on medication. The sample consisted of twenty young adults (mean age = 23.2, SD = 1.4, age range = 20–25 years, 5 females).

#### Procedure

The EEG measurement began with a 3-min relaxation phase [1.5 min with EC and 1.5 min with eyes open (EO)]. Instructions for the resting states were given on the computer display and were presented as follows: “A cross will be shown in the middle of the screen for a minute and a half. Please focus on the cross and relax” [for the EO condition] and “Keep your EC for a minute and a half and relax” [for the EC condition]. The rest phases were then followed by the auditory oddball task. The data of the task condition will not be presented here.

#### EEG recordings and analyses

The EEG was recorded from 58 Ag/AgCl electrodes using an elastic cap (Electrocap International) with a sampling rate of 500 Hz in a frequency band ranged between 0.5 and 100 Hz. The left mastoid was used as a reference and the right mastoid was recorded as an active channel. The data were also re-referenced off-line to an average of the left and right mastoids for further analysis. The electrodes were placed according to the international 10-10 system. For data analyses, only 21 electrode locations from the 10-20 system were used to avoid volume conduction effects between electrode sites located close together. Vertical and horizontal electrooculogram (EOG) was recorded for control of eye blinks and eye movements. The EEG recordings were high pass filtered at 1 Hz and corrected for eye movements using the Gratton and Coles algorithm (Gratton et al., 1983). Blink artifacts were rejected based on gradient criterion, i.e., maximal allowed voltage step (50 μ V), and difference criterion, i.e., maximal allowed absolute difference of two values in the segment (200 μ V), and also by visual inspection. The EEG was down-sampled to 250 Hz and segmented based on division in equal sized non-overlapping segments of 4096 ms length (1024 data points) for the rest intervals with EC and EO.

#### Spectral power and coherence, bispectrum and bicoherence

Spectral power was calculated using the fast Fourier transform (FFT) according to the following equation:

(2) $$X(f)=\frac{\sum_{k=0}^{N-1}X_{t}^{\prime }(k)\exp\left(\frac{-j2\pi kf}{f_{s}}\right)}{N}$$

where *N* is the number of frequency bins and *f*_*s*_ is the sampling rate. The FFT of the signals was calculated using 256 frequency bins with 0.98 Hz frequency resolution. We used a 128 point Blackman window, with 75% overlap. This procedure was also used for calculation of coherence and bispectral measures. Before calculation, EEG time series were normalized or adjusted to a zero mean value in order to exclude from analysis any signal offset arising from electrode half-cell potentials.

Spectral coherence between two time series at a certain frequency was calculated using the complex FFT according to the following equation:

(3) $$C_{XY}\left(f\right)=\frac{{\left|S_{XY}\left(f\right)\right|}^{2}}{S_{XX}\left(f\right)S_{YY}\left(f\right)}$$

where *S*_*XY*_ (*f*) = *S*_*X*_ (*f*) · S^*^_Y_ (*f*) is the cross-spectrum for the channels *X* and *Y*, and *S*_*XX*_ (*f*) and *S*_*YY*_ (*f*)are respective auto-spectra.

The single-sided *BIS* of a univariate time series was computed using the FFT-based method according to the following equation:

(4) $$BIS\left(f_{m},\;f_{n}\right)=\left|\langle X\left(f_{m}\right)\;\cdot \;X\left(f_{n}\right)\;\cdot \;X^{*}\left(f_{m}+f_{n}\right)\rangle \right|$$

where < · > denotes averaging or the expectation value, *X*(*f*) is the Fourier transform of the time series *X*_*t*_, and X^*^(*f*_1_ + *f*_2_) indicates the complex conjugate of *X*(*f*_1_ + *f*_2_). In the case of the *cBIS*, calculating CFC between two electrodes, the triple product is defined as:

(5) $$cBIS\left(f_{m},\;f_{n}\right)=\left|\langle X\left(f_{m}\right)\;\cdot \;Y\left(f_{n}\right)\;\cdot \;Y^{*}\left(f_{m}+f_{n}\right)\rangle \right|$$

When calculating *BIC* (CFC within one electrode or channel) or *cBIC* (CFC between two electrodes or channels), the *BIS* and the *cBIS* are normalized by the real triplet product:

(6) $$BIC\left(f_{m},\;f_{n}\right)=\frac{\left|\langle X\left(f_{m}\right)\;\cdot \;X\left(f_{n}\right)\;\cdot \;X^{*}\left(f_{m}+f_{n}\right)\rangle \right|}{\sqrt{\langle {\left|X\left(f_{m}\right)\;\cdot \;X\left(f_{n}\right)\right|}^{2}\;\cdot \;{\left|X\left(f_{m}+f_{n}\right)\right|}^{2}\rangle }}$$

and

(7) $$cBIC\left(f_{m},\;f_{n}\right)=\frac{\left|\langle X\left(f_{m}\right)\;\cdot \;Y\left(f_{n}\right)\;\cdot \;Y^{*}\left(f_{m}+f_{n}\right)\rangle \right|}{\sqrt{\langle {\left|X\left(f_{m}\right)\;\cdot \;Y\left(f_{n}\right)\right|}^{2}\;\cdot \;{\left|Y\left(f_{m}+f_{n}\right)\right|}^{2}\rangle }},$$

correspondingly.

It can be seen that *cBIS* and *cBIC* are asymmetric measures that means that *cBIS*_*XY*_(*f*_1_, *f*_2_) ≠ *cBIS*_*YX*_ (*f*_1_, *f*_2_) and also *cBIC*_*XY*_(*f*_1_, *f*_2_) ≠ *cBIC*_*YX*_(*f*_1_, *f*_2_). We used this property of the *cBIS* and *cBIC* to estimate directedness of the coupling, which is given when the information flow from one electrode to another electrode at the frequency *f*_1_ is much stronger than the information flow in the inverse direction at the frequency *f*_2_. If the information flow is similar or equal in both directions the coupling is defined as bidirectional.

In contrast to spectral power, (cross-)*BIS* depends like spectral coherence on both amplitude of the signal(s) and the degree of phase coupling between the frequencies, whereas *BIC* (*cBIC*) is a pure measure of the phase coupling.

#### Specific cross-frequency coupling measures based on the hilbert transform

As mentioned in the introduction, CFC can be at least of six different forms or types: (i) power to power, (ii) phase to phase, (iii) phase to power, (iv) power to frequency, (v) phase to frequency, and (vi) frequency to frequency. We present here only the first five CFC-measures, the frequency to frequency CFC will be omitted here. In the literature, different terms for CFC will be used in synonymous order, e.g., power to power = envelope-to-envelope = amplitude-to-amplitude or phase to power = phase-to-envelope = phase-to-amplitude etc. We decided to use the term “power” in all the cases to avoid misunderstandings or confusions.

To investigate these types of CFC, we used two different approaches: (i) based on calculation of PSI as described by Cohen (2008) for phase-amplitude coupling, and (ii) based on calculation of correlation coefficient between different cross-frequency components as described by Bruns and Eckhorn (2004) for envelope-to-envelope. Phase to phase CFC (or n:m phase synchronyzation) was calculated as described elsewhere (Tass et al., 1998; Rosenblum et al., 1999; Schack et al., 2005; Schack and Weiss, 2005; Witte et al., 2008). In addition, we extended the Cohen-algorithm for calculation of power to power CFC. All these algorithms were adapted and applied on the basis of Hilbert transform as described by Cohen (2008) and were calculated both within the signals (X or Y) and between them (XY).

At the first step, the epoch of the raw EEG data were band pass filtered in 16 different frequency ranges (fc [*f*_low_ − *f*_up_]: (1) 2 Hz [0.5–3.5 Hz]; (2) 3 Hz [1–5 Hz]; (3) 4 Hz [2–6 Hz]; (4) 5 Hz [3–7 Hz]; (5) 7 Hz [4.5–9.5 Hz]; (6) 8 Hz [5–11 Hz]; (7) 9 Hz [6–12 Hz]; (8) 10 Hz [6–14 Hz]; (9) 11 Hz [7–15 Hz]; (10) 12 Hz [8–16 Hz]; (11) 14 Hz [10–18 Hz]; (12) 18 Hz [14–22 Hz]; (13) 24 Hz [20–28 Hz]; (14) 28 Hz [24–32 Hz]; (15) 36 Hz [32–40 Hz]; (16) 70 Hz [65–75 Hz]) and then applied to the complex Hilbert transform. The instantaneous power and phase time series were extracted from the transformed data.

On the basis of instantaneous phases extracted from the Hilbert-transformed raw EEG signals given as: Φ_*X*_(*f*_*m*_, *t*) = arg[ϕ_*X*_(*f*_*m*_, *t*)] and Φ_*Y*_(*f*_*n*_, *t*) = arg[ϕ_*Y*_(*f*_*n*_, *t*)], correspondingly, the *n:m* phase synchronization between two oscillations at the center frequencies *f*_*m*_ and *f*_*n*_ were determined. The generalized phase difference (Δ Φ) according to *n* · *f*_*m*_ = *m* · *f*_*n*_ was calculated by:

(8) $$\begin{array}{l} \Delta \Phi_{X}\left(f_{m},\;f_{n},\;t\right)=n\;\cdot \;\Phi_{X}\left(f_{m},\;t\right)-m\;\cdot \;\Phi_{X}\left(f_{n},\;t\right),\\ \text{ mod}2\pi \;(\text{within the electrode}) \end{array}$$

(9) $$\begin{array}{l} \Delta \Phi_{XY}\left(f_{m},\;f_{n},\;t\right)=n\;\cdot \;\Phi_{X}\left(f_{m},\;t\right)-m\;\cdot \;\Phi_{Y}\left(f_{n},\;t\right),\\ \text{ mod}2\pi \;(\text{between the electrodes}) \end{array}$$

The *n:m PSI* was then defined by:

(10) $$\begin{array}{l} PSI_{X}\left(f_{m},\;f_{n},\;t\right)=\left|\langle e^{j\;\cdot \;\Delta \Phi_{X}\left(f_{m},\;f_{n},\;t\right)}\rangle \right|,\;j=\sqrt{-1}\\ \;(\text{within the electrode}) \end{array}$$

(11) $$\begin{array}{l} PSI_{XY}\left(f_{m},\;f_{n},\;t\right)=\left|\langle e^{j\;\cdot \;\Delta \Phi_{XY}\left(f_{m},\;f_{n},\;t\right)}\rangle \right|,\;j=\sqrt{-1}\\ \;(\text{between the electrodes}) \end{array}$$

where < · > denotes the averaging across *time*, in contrast to usual methods determining phase synchronization across *trials*.

At the second step, the power time series were normalized, detrended, or mean subtracted to remove DC-component and then also applied to the complex Hilbert transform. In this way, instantaneous phase of power time series was extracted. These time series were then used to determine power to power and phase to power coupling. For these purposes, PSI was calculated according to Equation 10 or 11 from instantaneous phases of power time series for calculation of the power to power CFC, and from instantaneous phase of the raw signal and instantaneous phase of normalized power time series, for calculation of the phase to power CFC (for details, see Cohen, 2008).

Power to power CFC was also investigated using correlation method (Bruns and Eckhorn, 2004; Witte et al., 2008). For this purpose, the correlation between the CFC components was calculated by the following equation:

(12) $$\rho_{X}^{\left(k\right)}\left(f_{m},\;f_{n},\;t\right)=\frac{\sum \left(A_{X}^{\left(k\right)}\left(f_{m},\;\tau \right)\;\cdot \;A_{Y}^{\left(k\right)}\left(f_{n},\;\tau \right)\right)}{\sqrt{E_{X}^{\left(k\right)}\left(f_{m},\;\tau \right)\;\cdot \;E_{Y}^{\left(k\right)}\left(f_{n},\;\tau \right)}},$$

where *k* is the number of data points in the segment, $A_{X}^{\left(k\right)}\left(f_{m},\;\tau \right)=a_{X}^{\left(k\right)}\left(f_{m},\;\tau \right)-\bar{a_{X}^{\left(k\right)}\left(f_{m},\;t\right)}$ and $A_{Y}^{\left(k\right)}\left(f_{n},\;\tau \right)=a_{Y}^{\left(k\right)}\left(f_{n},\;\tau \right)-\bar{a_{Y}^{\left(k\right)}\left(f_{n},\;t\right)}$ denote normalized CFC components of the signals *X* and *Y* at the center frequencies *f*_*m*_ and *f*_*n*_, correspondingly, and $E_{X}^{\left(k\right)}\left(f_{m},\;\tau \right)=\sum {\left(A_{X}^{\left(k\right)}\left(f_{m},\;\tau \right)\right)}^{2}$ and $E_{Y}^{\left(k\right)}\left(f_{n},\;\tau \right)=\sum {\left(A_{Y}^{\left(k\right)}\left(f_{n},\;\tau \right)\right)}^{2}$ are corresponding energies in the segment. Time series or CFC components were normalized by subtracting the ensemble or segments' means from corresponding instantaneous CFC-values. For determination of power to frequency and phase to frequency CFCs, *IF* of the bandpass-filtered signal component was determined using instantaneous phases as a derivative *IF*(*t*_*i*_) at the sample point *t*_*i*_ approximated by the difference equation:

(13) $$IF\left(f,\;t\right)≅\frac{IPh\left(f,\;t_{i+1}\right)-IPh\left(f,\;t_{i-1}\right)}{t_{i+1}-t_{i-1}}$$

where *IPh*(*f*, *t*_*i* + 1_) and *IPh*(*f*, *t*_*i* − 1_) are instantaneous phases of the signal at the time points *t*_*i* + 1_ and *t*_*i* − 1_, correspondingly. The different CFC components were applied to the Equation 12 to determine CFCs within and between corresponding time series.

All the CFC measures were determined across time within the segments and then averaged across segments. Before averaging, the measures (correlation coefficients and also PSIs) were normalized using Fisher-Z or tangent hyperbolicus transform:

(14) $$Z_{r}=\text{arctan }h\left(r\right)=\frac{1}{2}\text{ln}\left(\frac{1+r}{1-r}\right)$$

where *r* is the correlation coefficient or the PSI value.

#### Statistical evaluation

To determine whether CFC is greater than would be observed by chance, we used surrogate data test. For this purpose we generated surrogate data through a random permutation of phases of the time series (“phase shuffling”) of all EEG epochs at all considered channels and then calculated the corresponding synchronization measures between all possible electrode pairs of these surrogate data. Thereafter, we applied a bootstrapping procedure with 1000 resamples of the coupling measures gained from the surrogate data set and determined the threshold as the bootstrapping mean plus the confidence interval at a significance level of *p* < 0.0001. Only coupling values larger than the threshold value were considered for representation of data. In the case of simulated data, the threshold was determined in the same way with the difference that the surrogate data of the two simulated signals were generated 100 times.

For cross-frequency representations and statistical evaluation of the EEG data, we chose a most representative electrode pair (e.g., Fp1 and O2 in case of bispectral measures). In the case of spectral power and spectral coherence, we used the same electrodes (Fp1 and O2) to ensure the comparability. In the case of both spectral and bispectral measures, we divided the frequency spectrum into the four frequency bands: delta (2–4 Hz), theta (5–7 Hz), alpha1 (8–10 Hz), and alpha2 (11–13 Hz) and calculated power or coupling values within these frequency bands or between them. Spectral power was statistically evaluated using a Two-Way repeated measures ANOVA with two within-subject factors Eyes (EC and EO) and Electrodes (Fp1 and O2). Because the spectral coherence is a symmetrical measure, a One-Way repeated measures ANOVA with the within-subject factor Eyes (EC and EO) was calculated for the electrode pair Fp1-O2. A Two-Way repeated measures ANOVA with two within-subject factors Eyes (EC and EO) and Electrodes (Fp1 and O2) was calculated for *BIS* and bicoherence. In the case of *cBIS* and *cBIC* within the same frequency pairs (e.g., delta-delta, theta-theta, alpha1-alpha1, and alpha2-alpha2), we used in this ANOVA the factor Electrode Pair (Fp1-O2 and O2-Fp1) instead of Electrodes. For *cBIS* and *cBIC* between different frequencies (e.g., delta-theta, theta-alpha1, alpha1-alpha2, etc.), a Three-Way repeated measures ANOVA with three within-subject factors Eyes (EC and EO), Electrode Pair (Fp1-O2 and O2-Fp1) and Frequency (e.g., delta-theta vs. theta-delta) was used.

In the case of the specific CFC measures, separate ANOVAs were calculated for frequency components showing significant CFC in the corresponding grand averages. So, power to power CFC showed significant coupling in the frequency range between 5 and 14 Hz and concerns, above all, neighboring frequencies. We calculated then Two-Way repeated measures ANOVA with two within-subject factors Eyes and Frequency, where factor Frequency have had different levels: 7, 8, 9, 10, 11, and 12 Hz. We chose also a most representative electrode pair for corresponding CFCs. In the case of the power to power CFC, these electrodes were Fp1 and F3.

Greenhouse–Geisser epsilons were used in all ANOVAs for non-sphericity correction when necessary.

## Results

### Simulation data: illustration of two coupled oscillators

**Figure 3:**
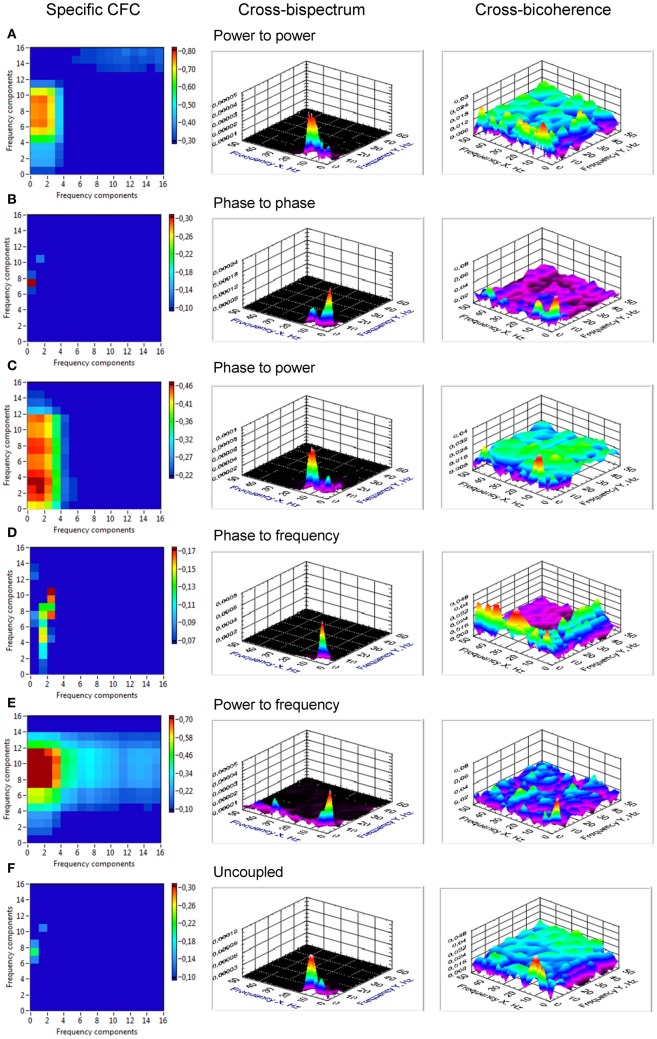
**Cross-frequency coupling in simulated data assessed using specific CFC-measures, cross-bispectrum, and cross-bicoherence. (A)** CFC for simulated signals with power to power modulation. The assessed coupling covers the CF interaction between 2 and 4 Hz of the low-frequency oscillator and 7 and 14 Hz of the high-frequency oscillator. Bispectrum and bicoherence show a peak for the 2–10 Hz CF interaction, which is more clearly seen in the bispectrum. **(B)** CFC for simulated signals with phase to phase modulation. The 2–10 Hz phase to phase relation is detected, as simulated in the data. Bispectrum and bicoherence also indicate this CF interaction but show also an additional delta-to-delta CFC peak. **(C)** CFC for simulated signals with phase to power modulation. The assessed coupling covers the CF interaction between 2 and 4 Hz of the low-frequency oscillator and 3 and 18 Hz of the high-frequency oscillator. Bispectrum and bicoherence indicate the 2–10 Hz CFC. **(D)** CFC for simulated signals with phase to frequency modulation. The CF interaction between the simulated frequencies (2 and 10 Hz) is shown. However, the CFC is higher for 3 to 7–11 Hz and 4 to 10–14 Hz CF interaction. Bispectrum and bicoherence showed a CFC between the low frequencies (between 1 and 8 Hz). Additionally, bicoherence showed a delta-beta CFC (2–5 to 24–28 Hz). **(E)** CFC for simulated signals with power to frequency modulation. The coupling is smeared between 2 and 5 Hz for low-frequency oscillator and 9 and 18 Hz for high-frequency oscillator. Bispectrum and bicoherence were not able to capture the power to frequency CF interactions and showed only peaks in the low-frequency range (1–4 Hz). **(F)** CFC for simulated uncoupled oscillators. Phase to phase CFC at the main simulated frequencies (2 and 10 Hz) is displayed indicating that the phase to phase relation that was initially fixed in the model system was not disturbed sufficiently by the present noise. Bispectrum indicates a CF interaction between 1 and 4 Hz and 8 and 14 Hz, and bicoherence—between 1 and 4 Hz and 10 and 13 Hz.

From Figure 2, it is apparent from the juxtaposition of the identical system simulated with and without noise that the effect of noise may either obscure or enhance certain types of CFC. For instance, the phase to power CFC shows itself clearly in absence of noise, but is difficult to recognize while noise is present (Figure 2C). The phase to phase CFC expresses itself clearer in the presence of noise (Figure 2B), because the two oscillator signals always return to the same phase coupling despite much variability otherwise. On the other hand, in absence of noise, there is no relative phase change after a transient, which renders an evaluation difficult and demands further investigation via CFC analysis. The CFC algorithms were, at first, applied to simulation data. The results of two coupled oscillators representing different types of CFCs are presented in Figure 3. The power to power CFC (Figure 3A) could be gathered with both specific CFC algorithms applied for this type of CFC (s. Methods for details). In both algorithms, there is smearing across frequencies, covering 2–4 Hz of the low-frequency oscillator and 7–14 Hz of the high-frequency oscillator (we present here only results calculated using the Cohen-algorithm). *BIS* and *BIC* showed a peak for the 2–10 Hz CF interaction. More clearly it can be seen in the bispectrum. Phase to phase CFC (Figure 3B) was registered more precisely indicating highest coupling for 2–10 Hz phase to phase relation, as simulated in the data. *BIS* and *BIC* also indicate this CF interaction but showed also an additional delta-to-delta CFC peak. Interestingly, the phase to phase coupling could be found not only for this type of CFC, but also for other types as well as in the case of uncoupled oscillators indicating that the phase to phase relation that was initially fixed in the model system was not disturbed sufficiently by the present noise. This issue is important and needs to be recognized: the CFC measures discussed here evaluate covariations of various orders, which are influenced by the degree of non-linearity and noise. The multifrequency behaviors and frequency smearing resulting from their influence are a real part of the signal and do not require corrections, since these phenomena are deviating from intuitive expectations based on linear oscillator theory (such as constant angular frequency across the range of phases for instance). The phase to power CFC (Figure 3C) has also been gathered with the algorithm applied for this type of CFC. There was also smearing across frequencies, covering 2–4 Hz of the low-frequency oscillator and 3–18 Hz of the high-frequency oscillator. *BIS* and *BIC* were also able to show the delta to alpha (2–10 Hz) CF interaction. In the case of phase to frequency CFC simulation (Figure 3D), there was indeed a significant CF interaction between the frequencies (2 and 10 Hz) but the CFC was higher for 3 to 7–11 Hz and 4 to 10–14 Hz CF interaction. Interestingly, phase to power CFC measure showed here strong coupling within the delta band (2–4 Hz) and also between delta und beta (14, 18, 24, and 28 Hz) frequencies. This coupling seems to be a byproduct of phase to frequency simulation. *BIS* and *BIC* showed a CFC between the low frequencies (between 1 and 8 Hz). Additionally, *BIC* showed a delta-beta CFC (2–5 to 24–28 Hz). The power to frequency CFC (Figure 3E) has also been gathered with the algorithm applied for this type of CFC but the coupling is highest at somewhat higher frequency components (14 and 18 Hz instead of 10 Hz). *BIS* and *BIC* were not able to capture this CFC and showed their peaks in the low frequency range between 1 and 4 Hz. The fact that *BIS* and *BIC* were not able to capture the phase to frequency and the power to frequency CF interactions is apparently due to the frequency modulations in the high-frequency signal disturbing the primary frequency ratio between the signals. Thus, these types of CF interactions can only be captured by specific CFC measures taking into account frequency modulations. In the case of the uncoupled oscillators (Figure 3F), there was a phase to phase CFC, as mentioned above, at the main simulated frequencies (2 and 10 Hz) but this coupling was not as strong as in the case of phase to phase simulation and showed also a slight smearing in the high frequency (9–11 Hz). In addition, *BIS* showed a CF interaction between 1–4 and 8–14 Hz, and *BIC*—between 1–4 and 10–13 Hz. In other words, there is CFC around the frequencies initially fixed in the model system that were not disturbed sufficiently by the present noise or noise provides a diffusion of CF relations.

### Resting state EEG with eyes closed and eyes open

**Figure 4:**
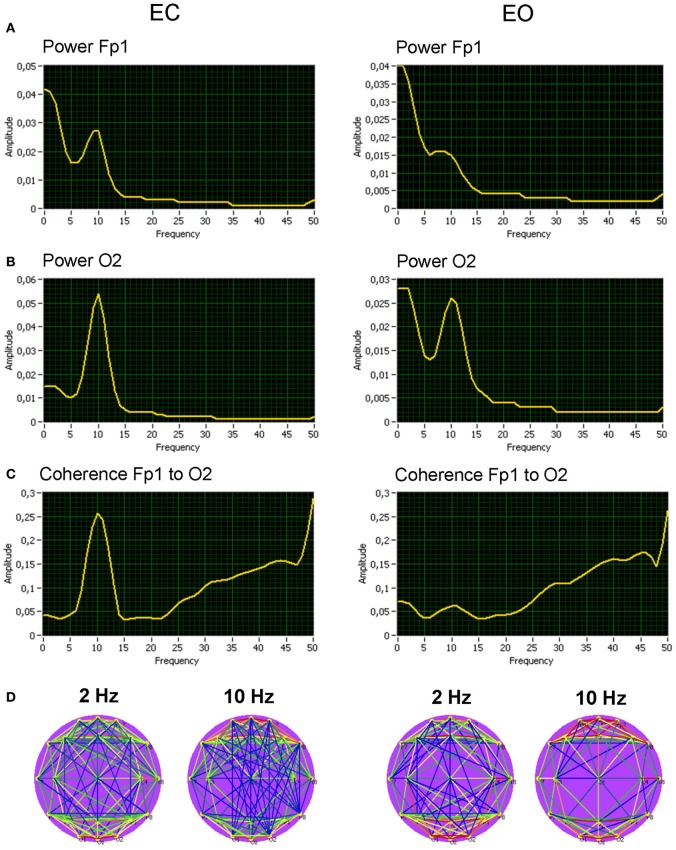
**Grand averages of the spectral power and coherence of the two selected electrodes, and corresponding coherence maps. (A)** Grand average of spectral power at the frontal electrode (Fp1). **(B)** Grand average of spectral power at the occipital electrode (O2). **(C)** Grand average of spectral coherence between the electrodes (Fp1 to O2). **(D)** Grand average of spectral coherence links above the threshold across all the electrodes for the delta (2 Hz) and the alpha (10 Hz) frequencies. All the diagrams and brain maps are displayed separately for eyes closed (EC) and open (EO). Clear 10-Hz peaks are displayed in spectral power and spectral coherence, which are stronger at occipital than at frontal site and stronger in EC than in EO condition. Brain maps display strong connections within frontal and occipital sites both for delta (2 Hz) and alpha (10 Hz) frequency.

We calculated at first spectral power and coherence to show that manipulation of the rest conditions (EC vs. EO) was in line with the literature about the resting state. Grand averages (across subjects) for the spectral power and coherence under the EC and the EO conditions are shown in Figure 4 for two selected electrodes (Fp1 and O2). As expected, the alpha spectral power was strongest at occipital than at frontal site [alpha1: *F*_(1, 19)_ = 24.7, *p* < 0.0001; alpha2: *F*_(1, 19)_ = 52.8, *p* < 0.0001] and stronger in EC than in EO condition [alpha1: *F*_(1, 19)_ = 51.5, *p* < 0.0001; alpha2: *F*_(1, 19)_ = 7.1, *p* < 0.05]. Statistical analyses showed also lower delta power in the EC as compared with EO condition [*F*_(1, 19)_ = 10.5, *p* < 0.01], above all at the occipital site [*F*_(1, 19)_ = 51.3, *p* < 0.0001]. In addition, coherence in the alpha frequency band was higher in the EC than in the EO condition [alpha1: *F*_(1, 19)_ = 15.9, *p* < 0.001; alpha2: *F*_(1, 19)_ = 13.7, *p* < 0.01].

### Bispectrum and bicoherence of the resting state EEG data

**Figure 5:**
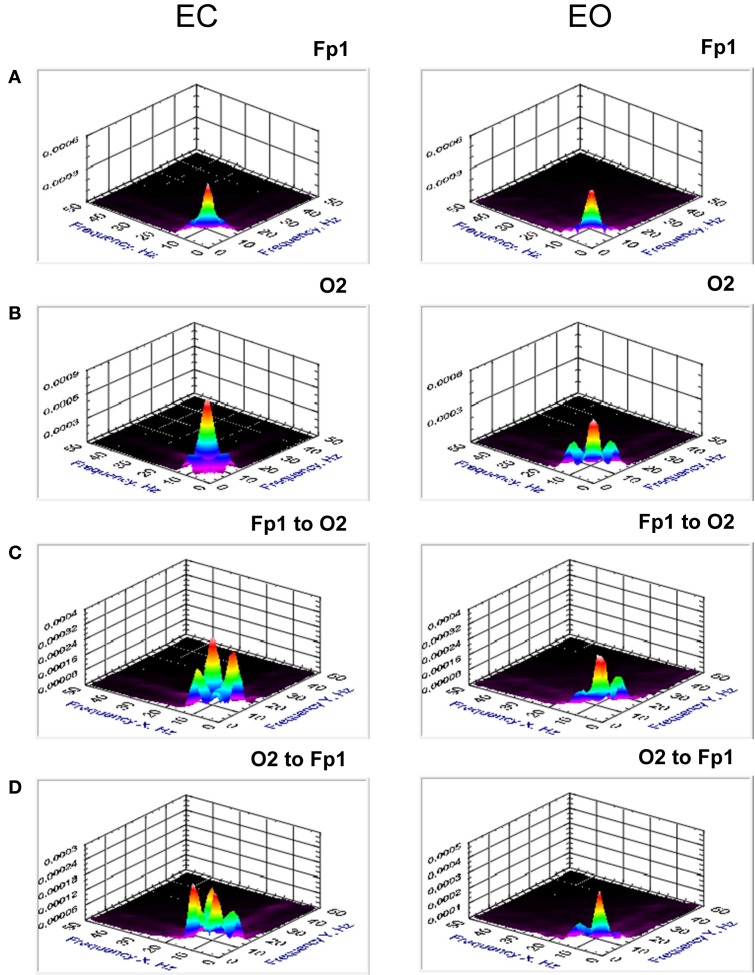
**Grand averages of the bispectrum at the selected electrodes (Fp1 and O2) and cross-bispectrum between them. (A)** Grand average of the bispectrum at the frontal electrode (Fp1). **(B)** Grand average of the bispectrum at the occipital electrode (O2). **(C)** Grand average of the cross-bispectrum between the electrodes (Fp1 to O2). **(D)** Grand average of the cross-bispectrum between the electrodes (O2 to Fp1). All the diagrams are displayed separately for eyes closed (EC) and open (EO). Bispectrum and also cross-bispectrum showed strong synchronization within the delta and alpha frequency bands as well as a CFC between them. The coupling is mostly stronger in EC than in EO condition.

**Figure 6:**
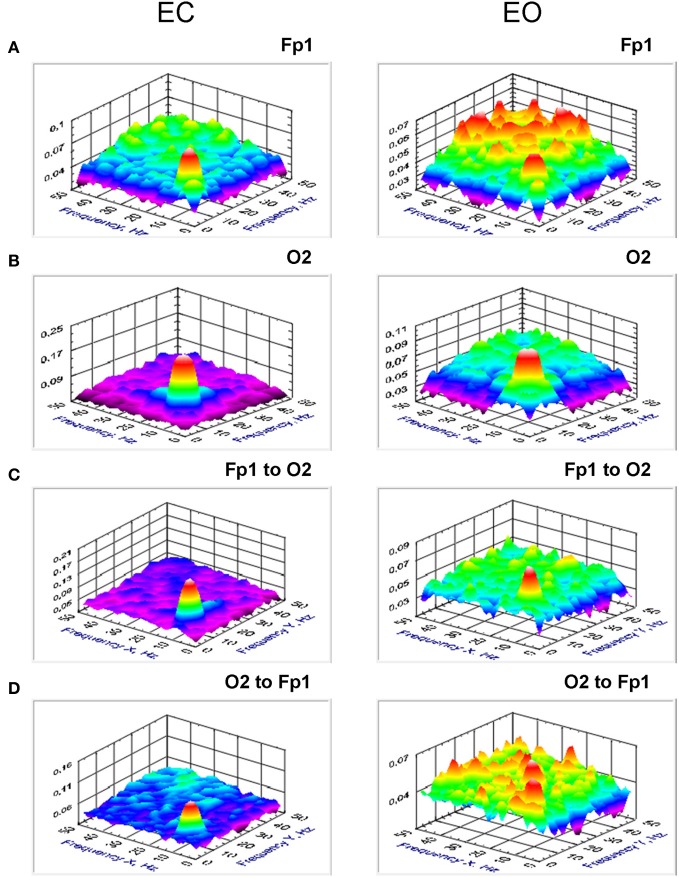
**Grand averages of the bicoherence at the selected electrodes (Fp1 and O2) and cross-bicoherence between them. (A)** Grand average of the bicoherence at the frontal electrode (Fp1). **(B)** Grand average of the bicoherence at the occipital electrode (O2). **(C)**. Grand average of the cross-bicoherence between the electrodes (Fp1 to O2). **(D)** Grand average of the cross-bicoherence between the electrodes (O2 to Fp1). All the diagrams are displayed separately for eyes closed (EC) and open (EO). Bicoherence and also cross-bicoherence showed strong coupling peak within the alpha frequency band. This coupling is mostly stronger in EC than in EO condition.

**Figure 7:**
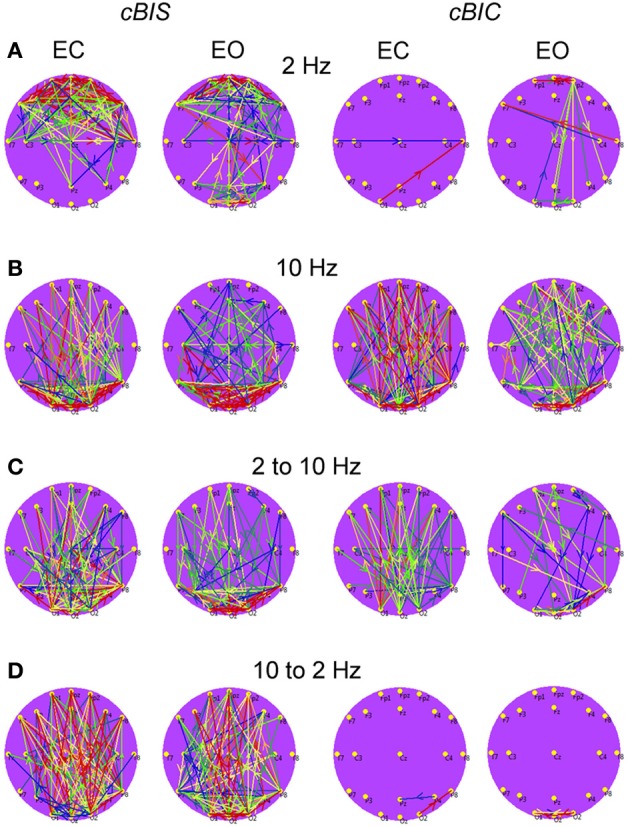
**Grand average brain maps of the cross-frequency and cross-electrode coupling within the same frequency and between the different frequencies. (A)** Grand average of the connections about the threshold at the frequency of 2 Hz. **(B)** Grand average of the connections about the threshold at the frequency of 10 Hz. **(C)** Grand average of the connections about the threshold for cross-frequencies from 2 to 10 Hz. **(D)** Grand average of the connections about the threshold for cross-frequencies from 10 to 2 Hz. All the diagrams are displayed separately for eyes closed (EC) and open (EO), and separately for cross-bispectrum (*cBIS*) and cross-bicoherence (*cBIC*). Blue color indicates low coupling and red color indicates high coupling. The arrows indicate the dominance or direction of the coupling. Strong larger-scale connections with predominantly posterior-to-anterior direction, especially in the case of 10 Hz or 10 to 2 Hz, are displayed. The direction of the coupling within the delta frequency (2 Hz) or in the case of the delta-to-alpha (2–10 Hz) CFC is inverse, especially in the EO condition, or mixed (anterior-to-posterior and posterior-to-anterior), especially in the EC condition.

Results (grand averages across subjects) of *BIS* and *BIC* for two electrodes (Fp1 and O2) as well as *cBIS* and *cBIC* between these electrodes are presented in Figures 5, 6. BIS and also cBIS showed strong synchronization within the delta and alpha frequency bands as well as a CFC between them. In contrast, *BIC* and also *cBIC* showed strong coupling peak in the alpha frequency band. Due to the fact that *cBIS* and *cBIC* are asymmetrical measures, we displayed corresponding diagrams for both the coupling from Fp1 to O2 and from O2 to Fp1. It can be seen that (a) the cross-electrode coupling patterns are different for the frontal-to-occipital (Fp1-O2) and occipital-to-frontal (O2-Fp1) directions, and (b) there is a strong asymmetry in the delta-alpha CFC, especially in the case of the *cBIS*, which is also different for these two pairs of electrodes and eyes conditions. In Figure 7, we mapped the significant connections between the electrodes within the delta (2 Hz) and the alpha (10 Hz) frequency bands and between them (2–10 and 10–2 Hz, separately) for *cBIS* and *cBIC* measures. The connection is, in this case, unidirectional if only one of the two connections is above the threshold and bidirectional if both connections are above the threshold. The threshold corresponds to the significance level determined using surrogate data (see Methods), if there are only few significant connections, in the other case, to maintain visibility, we displayed only about 30% of all significant connections. In comparison to simple coherence measure, there are very strong larger-scale connections with predominantly posterior-to-anterior direction, especially in the case of 10 Hz or 10–2 Hz. The direction of the coupling within the delta frequency (2 Hz) or in the case of the delta-to-alpha (2–10 Hz) CFC is inverse, especially in the EO condition, or mixed (anterior-to-posterior and posterior-to-anterior), especially in the EC condition.

The coupling within the alpha frequency was stronger during EC as compared to EO, at least for lower alpha [*BIS*: *F*_(1, 19)_ = 19.7, *p* < 0.001; *BIC*: *F*_(1, 19)_ = 21.7, *p* < 0.001; *cBIS*: *F*_(1, 19)_ = 27.5, *p* < 0.0001; *cBIC*: *F*_(1, 19)_ = 18.7, *p* < 0.001]. *cBIS* for these two electrodes (Fp1 to O2 and O2 to Fp1) in the delta frequency band was inversely higher in EO as compared with EC condition [*cBIS*: *F*_(1, 19)_ = 5.7, *p* < 0.05].

Statistical analyses of the *cBIS* for the different cross-frequency relations revealed significant interaction Cross-Electrode × Cross-Frequency for delta-alpha1 [*cBIS*: *F*_(1, 19)_ = 13.6, *p* < 0.01], delta-alpha2 [*cBIS*: *F*_(1, 19)_ = 26.8, *p* < 0.0001], theta-alpha1 [*cBIS*: *F*_(1, 19)_ = 9.1, *p* < 0.01], and theta-alpha2 [*cBIS*: *F*_(1, 19)_ = 13.3, *p* < 0.01] indicating stronger CFC from low to high frequency when going from frontal (Fp1) to occipital (O2) site and inversely stronger CFC from high to low frequency when going from occipital (O2) to frontal (Fp1) site. In the case of *cBIC*, as shown by significant interaction Eyes × Cross-Electrode × Cross-Freqeuncy for delta-alpha1 [*cBIC*: *F*_(1, 19)_ = 19.0, *p* < 0.001] and for delta-alpha2 [*cBIS*: *F*_(1, 19)_ = 6.1, *p* < 0.05] frequency relations, the direction of coupling is mostly from low to high frequency and is going from O2 to Fp1 in the EC condition and inverse in the EO condition. The CFC within the alpha frequency band (alpha1-to-alpha2) showed higher coupling in the EC condition than in the EO condition: *cBIS*: *F*_(1, 19)_ = 11.2, *p* < 0.01; *cBIC*: *F*_(1, 19)_ = 9.8, *p* < 0.01. The same is true also for theta-alpha CFC: theta-alpha1 [*cBIS*: *F*_(1, 19)_ = 7.0, *p* < 0.05; *cBIC*: *F*_(1, 19)_ = 4.4, *p* < 0.05] and theta-alpha2 [*cBIS*: *F*_(1, 19)_ = 9.9, *p* < 0.01; *cBIC*: *F*_(1, 19)_ = 11.8, *p* < 0.01].

### Resting state EEG captured using specific CFC measures

Power to power was determined using two different algorithms, which both gave similar results. We restrict our presentation to the algorithm based on calculation of *PSI* for amplitude-modulated signals (see Methods for details). Furthermore, there were no significant phase to frequency modulations.

**Figure 8:**
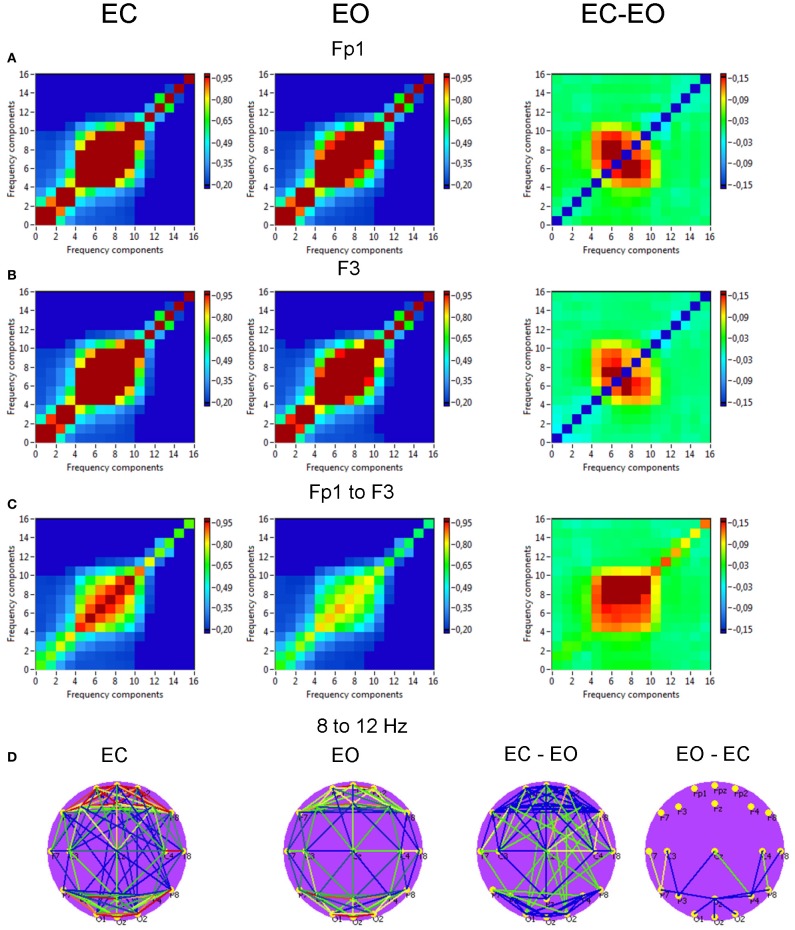
**Grand averages of the power to power cross-frequency coupling at the selected electrodes and between them. (A)** Grand average of the cross-frequency coupling at the electrode Fp1. **(B)** Grand average of the cross-frequency coupling at the electrode F3. **(C)** Grand average of the cross-frequency coupling between the electrodes Fp1 and F3. **(D)** Grand average brain maps with the connections about the threshold for cross-frequency coupling 8–12 Hz. All the diagrams and brain maps are displayed separately for eyes closed (EC) and open (EO), and separately for the difference between these conditions (EC-EO). In the brain maps, blue color indicates low coupling and red color indicates high coupling. Note: X- and Y-axes represent frequency components not the frequency bins. The strongest power to power CFC lays in the frequency range between 5 and 14 Hz and concerns, above all, neighboring frequencies, e.g., theta-to-alpha, alpha-to-alpha, and alpha-to-lower beta coupling. This coupling is mostly stronger in the EC condition than in the EO condition. Only few centro-parietal and parieto-occipital connections were stronger in the EO as compared to the EC condition.

*Power to power CFC* for two selected electrodes (Fp1 and F3) is displayed in Figure 8 and showed strong coupling between single frequencies within the delta, theta, alpha, and beta frequency bands; the strongest CFC lay in the frequency range between 5 and 14 Hz and concerns, above all, neighboring frequencies, e.g., theta-to-alpha, alpha-to-alpha, and alpha-to-lower beta coupling. This coupling between the selected electrodes (Fp1 to F3) was also higher in the EC as compared with EO condition: theta-alpha [7 to 8–12 Hz: *F*_(1, 19)_ = 10.2, *p* < 0.01] and alpha-alpha [8 to 9–12 Hz: *F*_(1, 19)_ = 9.4, *p* < 0.01]. The brain maps showed strong connections (e.g., 8–12 Hz coupling) within anterior and posterior regions but large-scale connections are attenuated (compare also Bruns and Eckhorn, 2004). Due to the fact that the coupling between the electrodes was mostly bidirectional, the arrows are omitted in the brain maps. It is also visible that the coupling in the EC condition is mostly stronger than in the EO condition, and that only few centro-parietal and parieto-occipital connections were stronger in the EO as compared to the EC condition.

**Figure 9:**
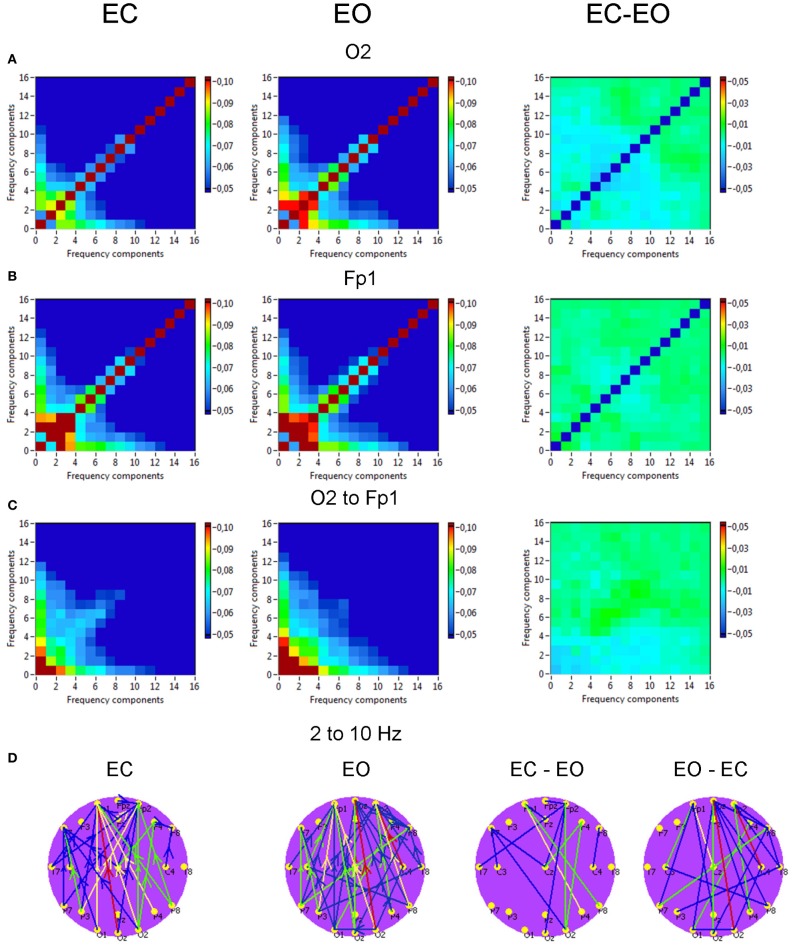
**Grand averages of the phase to phase cross-frequency coupling at the selected electrodes and between them. (A)** Grand average of the cross-frequency coupling at the electrode O2. **(B)** Grand average of the cross-frequency coupling at the electrode Fp1. **(C)** Grand average of the cross-frequency coupling between the electrodes O2 and Fp1. **(D)** Grand average of the connections about the threshold for cross-frequencies coupling 2–10 Hz. All the diagrams and brain maps are displayed separately for eyes closed (EC) and open (EO), and separately for the difference between these conditions (EC-EO). In the brain maps **(D)**, blue color indicates low coupling and red color indicates high coupling. The arrows indicate the dominance or direction of the coupling. Note: X- and Y-axes represent frequency components not the frequency bins. The coupling is strongest within and between delta and theta frequencies. The CFC between delta and alpha frequencies is moderate and related to connections going from posterior to anterior regions.

*Phase to phase CFC* for electrodes O2 and Fp1 is presented in Figure 9 and was strongest within and between delta and theta frequencies. The CFC between delta and alpha frequencies was moderate and also related to connections going from posterior to anterior. For the selected pair of electrodes, there were no significant differences between the resting state conditions (EC vs. EO). The difference brain maps (EC-EO and EO-EC) showed that, at least the half of connections were stronger in the EO condition than in the EC condition.

**Figure 10:**
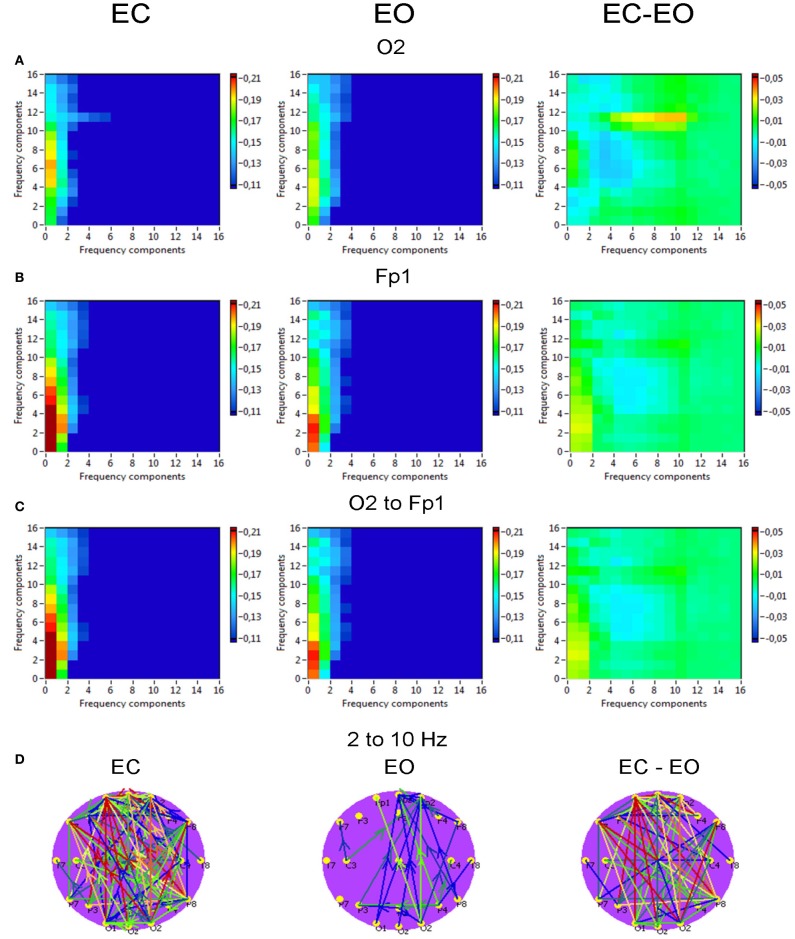
**Grand averages of the phase to power cross-frequency coupling at the selected electrodes and between them. (A)** Grand average of the cross-frequency coupling at the electrode O2. **(B)** Grand average of the cross-frequency coupling at the electrode Fp1. **(C)** Grand average of the cross-frequency coupling between the electrodes O2 and Fp1. **(D)** Grand average of the connections about the threshold for cross-frequencies coupling 2–10 Hz. All the diagrams and brain maps are displayed separately for eyes closed (EC) and open (EO), and separately for the difference between these conditions (EC-EO). In the brain maps, blue color indicates low coupling and red color indicates high coupling. The arrows indicate the dominance or direction of the coupling. Note: X- and Y-axes represent frequency components not the frequency bins. This CFC is related only to the delta phase, which was coupled with amplitude modulations in the other higher frequency bands. Brain maps for 2–10 Hz CFC display larger-scale coupling, which is strongest when going from posterior to anterior brain regions. These connections are stronger in the EC than in the EO condition.

*Phase to power CFC* for electrodes O2 and Fp1 is displayed in Figure 10. This CFC was related only to the delta phase, which was coupled with amplitude modulations in the other higher frequency bands. Brain maps for 2–10 Hz CFC showed that this mostly larger-scale coupling is strongest when going from posterior to anterior brain regions. For the selected pair of electrodes, the difference between the resting state conditions (EC vs. EO) was not significant. On the other hand, as depicted in the difference brain map (EC-EO), all connections in the EC condition were stronger than in the EO condition.

**Figure 11:**
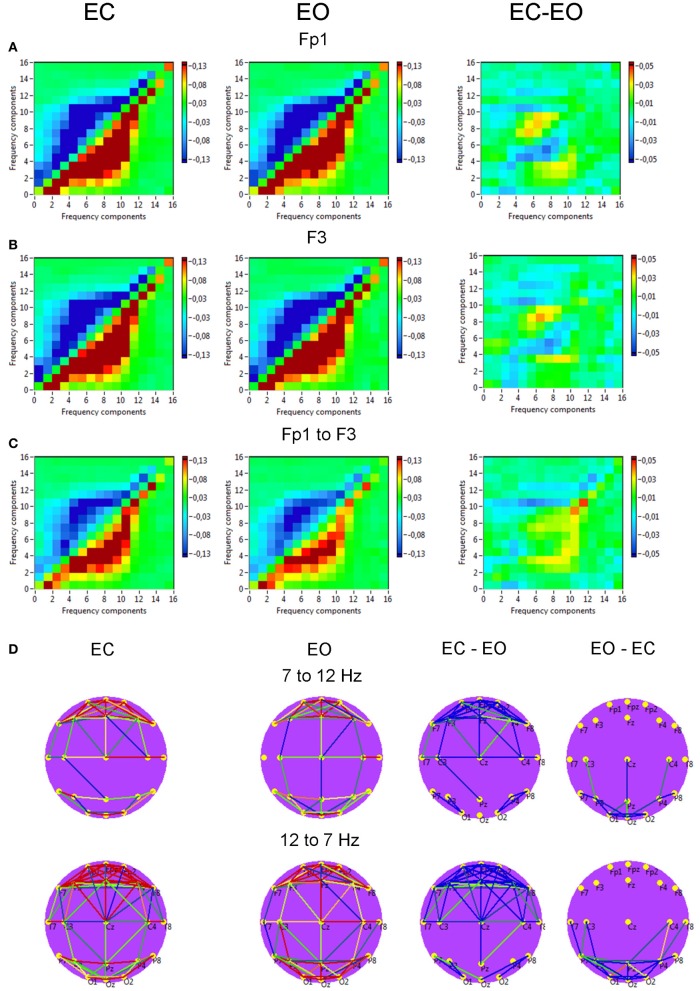
**Grand averages of the power to frequency cross-frequency coupling at the selected electrodes and between them. (A)** Grand average of the cross-frequency coupling at the electrode Fp1. **(B)** Grand average of the cross-frequency coupling at the electrode F3. **(C)** Grand average of the cross-frequency coupling between the electrodes Fp1 and F3. **(D)** Grand average brain maps with the connections about the threshold for cross-frequency coupling 7–12 Hz. All the diagrams and brain maps are displayed separately for eyes closed (EC) and open (EO), and separately for the difference between these conditions (EC-EO). In the brain maps, blue color indicates low coupling and red color indicates high coupling. Due to the fact that the coupling between the electrodes was mostly bidirectional, the arrows are omitted. Note: X- and Y-axes represent frequency components not the frequency bins. The strongest CFC lays in the frequency range between 3 and 14 Hz and concerns, above all, neighboring frequencies, e.g., theta-to-alpha, alpha-to-alpha, and alpha-to-lower beta coupling. The coupling switches their polarity dependent on frequency components: power to frequency coupling is positive when modulating frequency is higher than modulated frequency and negative when modulating frequency is lower than modulated frequency.

*Power to frequency CFC* for two selected electrodes (Fp1 and F3) is displayed in Figure 11 and showed strong coupling between single frequencies within the delta, theta, alpha, and beta frequency bands; the strongest CFC lay in the frequency range between 3 and 14 Hz and concerns, above all, neighboring frequencies, e.g., theta-to-alpha, alpha-to-alpha, and alpha-to-lower beta coupling. In addition, power to frequency modulations switch their polarity dependent on frequency components: power to frequency coupling is positive when modulating frequency is higher than modulated frequency and negative when modulating frequency is lower than modulated frequency. The brain maps showed strong negative (e.g., 7–12 Hz coupling) and positive (e.g., 12–7 Hz coupling) connections within anterior and posterior regions but large-scale connections are attenuated. Due to the fact that the coupling between the electrodes was mostly bidirectional, the arrows are omitted in the brain maps. The topology of negative and positive coupling between these two frequencies is similar. Probably, processes underlying these two different modulations (7 Hz power to 12 Hz frequency modulation and 12 Hz power to 7 Hz frequency modulation) are the same. It is also visible that the coupling in the EC condition is stronger than in the EO condition at frontal sites, and that centro-parietal and parieto-occipital connections were stronger in the EO as compared to the EC condition. The statistical analysis of the coupling between the selected electrodes (Fp1 to F3) showed no significant differences between EC and EO conditions.

## Discussion

The aim of the study was to introduce and to test different CFC measures on the simulated and the resting state EEG data. Our results showed that the CFC-measures mostly correctly detect the nature of CFC in the simulated data and display different coupling dynamics in the experimental EEG data. Our resting state data showed delta-alpha CFC in terms of *cBIS* and *cBIC* as well as other specific CFC measures (e.g., phase to phase or phase to amplitude). This coupling, which was generally higher in the EC than in the EO condition, was mostly located within the frontal and the parieto-occipital regions, and most important these regions were connected through lager-scale coupling with different coupling direction (anterior to posterior or inverse).

### CFC of simulated data

We generated oscillatory time series from non-linearly coupled dynamic systems whose mathematical skeleton was derived from large-scale brain network equations, but its parameters were freely chosen to maximize the effects of CFC. We systematically modeled and simulated the various scenarios of CFC under the influence of noise to obtain biologically realistic oscillator dynamics. We successfully showed that (i) specific CFC-measures mostly correctly detect the nature of CFC under noise conditions, (ii) *BIS* and *BIC* also detected the delta-to-alpha CFC in simulated data. In conjunction, these two sets of measures hence provide a powerful toolbox to reveal the nature of couplings from experimental data.

### Resting state with eyes closed and eyes open

Using different CFC measures, we found cross-frequency modulations concerning amplitude, phase, and frequency changes in the EEG signals during rest. The strongest CFC during rest both with EO and EC was found within and between the delta and the alpha frequency bands but also theta and beta frequencies were involved into cross-frequency interactions. Different CFC measures showed different cross-frequency synchronization or coupling patterns indicating that different neural mechanisms are at work. Power to power modulations indicate CFC between closer frequencies within the delta, theta, and alpha frequency bands. This coupling was also mostly symmetric or bidirectional. As for power to power modulations, also power to frequency CFC was found to be strong within the delta, theta and especially alpha frequency band but in contrast to former power to frequency modulations were broader and include beside delta-to-theta and theta-to-alpha also delta-to-alpha modulations. In addition, power to frequency modulations switch their polarity dependent on frequency components: power to frequency coupling is positive when modulating frequency is higher than modulated frequency and negative when modulating frequency is lower than modulated frequency. We showed that the topology of negative and positive coupling between two different frequencies (e.g., 7–12 Hz or 12–7 Hz) is similar. This finding leaves us to believe that processes underlying these two different modulations (7 Hz power to 12 Hz frequency modulation and 12 Hz power to 7 Hz frequency modulation) may probably be the same, or at least of the same nature. As for power to power CFC, power to frequency CFC is mostly bidirectional and short-range. Thus, CFC measures are able not only to describe the long-range synchronization or coupling but complete our understanding of short-range coupling involved in local networks.

Interestingly, *BIS* which is considered in the literature as a pure amplitude CFC measure was able to detect the delta-alpha relations that were absent in the specific power to power measure but could be found using power-to-phase or phase to phase measures. In addition, the coupling found using bispectral analyses was asymmetric, i.e., there was directionality in the coupling. Interestingly, the coupling found by *BIC* was very strong within the alpha band (like the amplitude-to-amplitude CFC) and moderate or even absent regarding the delta-alpha relations. *BIS* and *BIC* measure, in contrast to specific measures, the non-linear quadratic coupling, whereas the relation between amplitude and phase in this coupling is not always clear. The fact that we found clear delta-alpha *BIS* peak in the phase to phase simulated data confirms our statement: *BIS* reflects not only amplitude but also phase modulations. Whether the power to frequency CFC plays here a role and what its influence is that remains to be seen.

Another interesting point is that delta-alpha CFC is above all related to large-scale connections going from anterior to posterior in the case of 2 Hz modulation of alpha and is rather inverse if CFC modulation is 10–2 Hz as shown in the *cBIS* and partially in the *cBIC*. Interestingly, if in the case of 10–2 Hz frequency modulation, posterior-to-anterior CFC is predominantly in both EC and EO conditions, in the case of 2–10 Hz frequency modulation, the CFC is anterior-to-posterior in the EO condition but rather inverse or mixed (posterior-to-anterior and anterior-to-posterior) in the EC condition. This different direction of the delta-alpha (or alpha-delta) CFC is apparently contingent on the locations of delta and alpha generators. It is well–known that delta oscillations are generated anterior, whereas alpha oscillations have posterior and also anterior origin (Michel et al., 1992; Tsuno et al., 2002; Canuet et al., 2011). Apart from the direction of the coupling, localization of delta and alpha frequency generators at anterior and posterior sites allows the explanation of the larger-scale coupling, which is detectable using different CFC methods, especially if alpha oscillations are involved. Interestingly, this larger-scale coupling was found also within the alpha frequency, and this coupling has predominantly posterior-to-anterior direction indicating the influence of the alpha-frequency generators on the other brain regions. This larger-scale coupling is especially strong in the EC condition, when alpha oscillations are much more pronounced, and may be related to the inhibitory function of alpha oscillations reported in the literature (Klimesch et al., 2007; Jensen and Mazaheri, 2010; Mathewson et al., 2011).

In addition, we also found differences in the CFC when compared EC with EO conditions. Mostly, CFC was stronger in the EC condition as compared with EO condition. Decrease in spectral alpha power (also called alpha depression) during rest with EO compared to rest with EC, also shown in our study, is a well-known phenomenon (see Klimesch, 1999, for a review). Normally, alpha depression in the EO condition is associated with brain activation caused by increased external stimulation through the opening of the eyes or visual input (Klimesch, 1999). It can be seen that this brain activation through opening the eyes reduces also spectral coherence and CFC, and evokes different CFC patterns between the different electrode sites and frequencies indicating that there are different processes at work. Besides the long-range connectivity also short-range connectivity reduces in general its strength and alters its topology through opening the eyes. Interestingly, the short-range connectivity, as shown by power to power and power to frequency CFC, is higher in EC than in EO above all frontally but higher in EO than in EC parieto-occipital indicating higher segregation during EC at frontal sites and higher segregation during EO at parieto-occipital sites. These short-range CFC patterns probably describing local synchronization complement our knowledge about the local networks, which are usually delineated by synchronization at single frequencies.

### Methodological: advantage of using CFC measures and its limitations

The interaction between different frequencies investigated here adds another dimension in understanding complex neural dynamics of the frequency-specific neuronal networks. Neuronal cell assemblies oscillating synchronously at different frequencies provide an efficient basis for integrative processes in the brain (Buzsáki and Draguhn, 2004). Separate cell assemblies communicate with each other to integrate single information flows into a common network. Non-linear dynamic system theory teaches us that time-scale separation, that is frequency separation in this context, offers a natural means in non-linear systems to separate information flows. Then CFC, allowing accurate timing between different oscillatory rhythms, may be one of the mechanisms underlying the re-integration of these separated information flows, or, said differently, allowing for a communication between different cell assemblies (Klimesch et al., 2008; Sauseng et al., 2008; Canolty et al., 2010). As we have shown, CFC measures (especially *BIS* and *BIC*) in comparison to the classical coherence measure describe well the large-scale coupling. Due to the fact that *BIS* and *BIC* as well as the phase to amplitude CFC measure are asymmetric, they can provide the information about the coupling directionality, even though they make no statement about causality that is the direction of the information flow. In addition, *BIS* and *BIC* reflect non-linear coupling between different oscillations both within and between the different electrodes. Here, we have clearly shown that the different CFC measures provide different insights about the cross-frequency interaction. These outcomes or synchronization patterns should be considered not as alternative but rather as complementary to each other. All these interaction patterns found by different CFC measures exist simultaneously in biological signals (including neuroimaging signals such as neuroelectric or neuromagnetic measurements) and thus give us a more complete picture about information processing in the brain.

### Theoretical: significance of delta and alpha interactions

As reported earlier, Isler et al. (2008) found CF delta-alpha modulations in terms of *BIC* in widespread fronto-central, right parietal, temporal, and occipital regions, and also between them. This CFC found in an auditory novelty oddball task was interpreted as a neural mechanism for the orienting response. In the study of (Cohen et al., 2009a), delta-alpha phase to amplitude CFC found in a competitive decision-making task was suggested to reflect a coding mechanism of feedback valence information. Our resting state data showed delta-alpha (and also alpha-delta) CFC in terms of (cross-) *BIS* and (cross-) *BIC* as well as other specific CFC measures (e.g., phase to phase or phase to amplitude). This coupling is mostly located within the frontal and the parieto-occipital regions, and most important these regions are connected through lager-scale coupling providing direct communication between different cell assemblies located in these regions. Thereby, this coupling is asymmetric mostly from the parieto-occipital to frontal regions, especially during rest with EC, whereas in the rest condition with EO the direction of coupling, especially delta-to-alpha (2–10 Hz) can be inverse. As mentioned above, delta and alpha oscillations have different origin: whereas delta oscillations are generated anterior, alpha oscillations have posterior and, to some extent, also anterior origin (Michel et al., 1992; Tsuno et al., 2002; Canuet et al., 2011). Following Steriade and Timofeev (2003), delta oscillations are generated by neocortical and thalamo-cortical networks. Enhanced oscillatory activity in the delta frequency range during cognitive tasks is often considered as an indicator of attentional task demands (Harmony et al., 1996; McEvoy et al., 2001) and of syntactic language processing (Roehm et al., 2004). Strong or synchronized alpha activity is associated with cortical deactivation or inhibition, whereas strongly desynchronized alpha activity reflects a state of high excitability (Klimesch, 1999; Klimesch et al., 2007, 2008). Larger amplitudes of synchronized alpha activity typical for rest state with EC are associated with a brain state of reduced information processing (Pfurtscheller and Lopes da Silva, 1999; Pfurtscheller, 2001) and are consistent with the concepts of “idling” or “nil working” (Adrian and Matthews, 1934). Alpha activity covers a wide range of different cognitive functions and is strongly involved in memory processes, whereby pronounced ERS (event-related synchronization) was observed during retention but strong ERD (event-related desynchronization) during retrival (Klimesch, 1999; Jensen et al., 2002; Schack and Klimesch, 2002; Sauseng et al., 2005; Klimesch et al., 2007). The envelopes of various frequency bands of neuroelectric activity are correlated with the hemodynamic signals as measured in Bold fMRI giving rise to ultraslow intermittent spontaneous coherent fluctuations in the absence of an explicit task (Biswal et al., 1995; Greicius et al., 2003; Müller et al., 2003a,b; Damoiseaux et al., 2006; Deco et al., 2009; Venables et al., 2009). Large-scale brain modeling efforts demonstrated the stochastic nature of the spatiotemporal fluctuations (Deco et al., 2008, 2009; Ghosh et al., 2008). The delta-alpha CFC found in our study during resting state allows supposing that this CF interaction capturing the intrinsic network dynamics might play a crucial rule in information exchange and its integration. Furthermore, there is neurophysiological evidence that resting-state networks undergo profound reorganization from childhood to old age (Müller and Lindenberger, 2012).

### Conflict of interest statement

The authors declare that the research was conducted in the absence of any commercial or financial relationships that could be construed as a potential conflict of interest.
